# On semidiscrete constant mean curvature surfaces and their associated families

**DOI:** 10.1007/s00605-016-0929-6

**Published:** 2016-05-23

**Authors:** Wolfgang Carl

**Affiliations:** Institute of Geometry, TU Graz, Kopernikusgasse 24, 8010 Graz, Austria

**Keywords:** Semidiscrete surface, Constant mean curvature, Associated family, Weierstrass representation, Lax pair representation, 53A05, 53A10, 39A12

## Abstract

The present paper studies semidiscrete surfaces in three-dimensional Euclidean space within the framework of integrable systems. In particular, we investigate semidiscrete surfaces with constant mean curvature along with their associated families. The notion of mean curvature introduced in this paper is motivated by a recently developed curvature theory for quadrilateral meshes equipped with unit normal vectors at the vertices, and extends previous work on semidiscrete surfaces. In the situation of vanishing mean curvature, the associated families are defined via a Weierstrass representation. For the general cmc case, we introduce a Lax pair representation that directly defines associated families of cmc surfaces, and is connected to a semidiscrete $\sinh$-Gordon equation. Utilizing this theory we investigate semidiscrete Delaunay surfaces and their connection to elliptic billiards.

## Introduction

Surfaces with constant mean curvature *H* or constant Gauss curvature *K* have been of particular interest in differential geometry for a long time. In a modern viewpoint, these special geometries are associated with the theory of integrable systems, not least due to rather recent developments in discrete differential geometry (cf. Bobenko and Suris [7]). Typically the investigation of constant curvature surfaces is tied to specific parametrizations, like isothermic parametrizations for constant mean curvature surfaces.

Over the last decades, various discrete versions of these special parametrizations have been established. For a comprehensive overview see Bobenko and Pinkall [5] or Bobenko and Suris [7]. Generally, different kinds of parametrizations (conjugate, asymptotic,...) have their own way of discretization. For this reason, discretizing entire families of smooth surfaces is a challenge, if the type of parametrization changes. Accordingly, a unifying discrete curvature theory is still an active topic of research. As a first step toward this direction, Bobenko et al. [6] introduced a general curvature theory for polyhedral meshes with planar faces based on mesh parallelity. Their theory is capable of unifying notable previously defined classes of surfaces, such as discrete isothermic minimal or constant mean curvature surfaces. More recently, Hoffmann et al. [12] presented a discrete parametrized surface theory for quadrilateral meshes equipped with unit normal vectors at the vertices, permitting non-planar faces. Their theory encompasses a remarkably large class of existing discrete special parametrizations. In addition it provides a deeper insight into the associated families of discrete constant curvature surfaces.

For semidiscrete surfaces, represented by parametrizations possessing one discrete variable and one continuous variable, the situation is quite similar to the discrete case. The analysis of semidiscrete surfaces with $H=\text {const.}$ respectively $K=\text {const.}$ is bound to isothermic resp. asymptotic parametrizations (cf. Müller and Wallner [16], Müller [15], Burstall et al. [8], and Wallner [23]). However, to the author’s knowledge, results concerning their associated families have been missing so far.

### Outline and results

In the present paper we investigate two distinct situations: (i) semidiscrete surfaces with vanishing mean curvature (*minimal surfaces*), and (ii) semidiscrete surfaces with constant but non-vanishing mean curvature (*cmc surfaces*). Since we are especially interested in the associated families of these surfaces, we do not restrict ourselves to isothermic parametrizations. Thus, at the beginning (see Sect. 2), we translate the discrete curvature theory introduced by Hoffmann et al. [12] to the semidiscrete setting. We also highlight the intersection with the curvature theory for semidiscrete conjugate parametrizations previously considered by Karpenkov and Wallner [13].

In Sect. 3, we recapitulate the notion of isothermic parametrizations. In particular, we show that a semidiscrete surface is isothermic if and only if its quaternionic cross ratio allows for a specific factorization (cf. Lemma 5).

Subsequently, in Sect. 4, we investigate semidiscrete isothermic minimal surfaces. Their Weierstrass representation, established by Rossman and Yasumoto [20], immediately gives rise to their associated families, whose members are however no longer isothermic. The main result of this section is that all the members of these associated families are minimal as well (cf. Theorem 1). Moreover, we show that the conjugate surface of an isothermic minimal surface is asymptotically parametrized.

In Sect. 5, we introduce a Lax pair representation for semidiscrete isothermic cmc surfaces, which directly contains the definition of their associated families. We prove that the members of these associated families, which again are no longer isothermic, all have the same constant mean curvature (cf. Theorem 2).

We conclude the paper by investigating the Lax pair representation of semidiscrete rotational symmetric cmc surfaces (see Sect. 6). It turns out that the discrete versions of the classical Delaunay rolling ellipse construction described by Hoffmann [11] and Bobenko et al. [6] also apply to the semidiscrete setting.

## A curvature theory for semidiscrete surfaces

Our main object of study are two-dimensional semidiscrete surfaces in three-dimensional Euclidean space represented by parametrizations

$$\begin{aligned} x:\mathbb {Z}\times \mathbb {R}\supseteq D\rightarrow \mathbb {R}^3:(k,t)\mapsto x(k,t) \end{aligned}$$

possessing one discrete variable and one continuous variable. Throughout this paper we assume that *x* is at least once continuously differentiable w.r.t. the second argument. We abbreviate the corresponding derivative by $\partial x$. The forward difference w.r.t. the discrete parameter is denoted by

$$\begin{aligned} \Delta x:=x_1-x, \end{aligned}$$

where the notation $x_1$ indicates an index shift: $x_1(k,t):=x(k+1,t)$. We only consider *regular* semidiscrete surfaces having the property that the sets $\{\Delta x,\partial x\}$, $\{\Delta x,\partial x_1\}$, and $\{\Delta x, \partial x+\partial x_1\}$ are linearly independent throughout.

Just like a smooth parametrized surface can be viewed as built of its *contact elements* (consisting of a point together with the surface normal at this point), we henceforth consider a semidiscrete surface to be represented by a *pair* of weakly coupled parametrizations. Translating the relation between a surface and its Gauss map to the semidiscrete setting, we define:

### Definition 1

A pair of semidiscrete surfaces $(x,n):\mathbb {Z}\times \mathbb {R}\supseteq D\rightarrow \mathbb {R}^3\times \mathbb {S}^2$ is called *coupled*, if

1 $$\begin{aligned} \Delta x\perp (n+n_1)\quad \text {and}\quad \partial x\perp n \end{aligned}$$

throughout the parameter domain.

The following definition contains a (partial) limit version of the “midpoint connectors” of a quadrilateral considered by Hoffmann et al. [12] as replacements of the first order partial derivatives of a smooth parametrization.

### Definition 2

For a semidiscrete surface (*x*, *n*) we define the *partial derivatives*

$$\begin{aligned} \partial _1 x:=\Delta x=x_1-x,\quad \partial _2 x:=\frac{\partial x+\partial x_1}{2}, \end{aligned}$$

as well as the *strip normal*

$$\begin{aligned} N:=\frac{\partial _1 n\times \partial _2 n}{\Vert \partial _1 n\times \partial _2 n\Vert }=\frac{\Delta n\times (\partial n+\partial n_1)}{\Vert \Delta n\times (\partial n+\partial n_1)\Vert }. \end{aligned}$$

In classical surface theory the principal curvatures of a surface at a point are defined as the eigenvalues of the shape operator that lives on the tangent plane at this point. In the semidiscrete case the fundamental forms and the shape operator live on the plane perpendicular to the strip normal *N*.

### Definition 3

Let (*x*, *n*) be a semidiscrete surface with strip normal *N* and let $\pi$ denote the orthogonal projection onto the plane perpendicular to *N*, i.e., $\pi (x):=x-\langle x,N\rangle N$. Mimicking the smooth case, we define the *fundamental forms* ${\text {I}}$, ${\text {II}}$, ${\text {III}}$, and the *shape operator* *S* by

$$\begin{aligned} {\text {I}}&:=\begin{pmatrix} \Vert \pi (\partial _1 x)\Vert ^2 &{}\quad \langle \pi (\partial _1 x), \pi (\partial _2 x)\rangle \\ \text {symm.} &{}\quad \Vert \pi (\partial _2 x)\Vert ^2 \end{pmatrix},&{\text {III}}&:=\begin{pmatrix} \Vert \partial _1 n\Vert ^2 &{}\quad \langle \partial _1 n, \partial _2 n\rangle \\ \text {symm.} &{}\quad \Vert \partial _2 n\Vert ^2 \end{pmatrix},\\ {\text {II}}&:=-\begin{pmatrix} \langle \partial _1 x, \partial _1 n\rangle &{}\quad \langle \partial _1 x, \partial _2 n\rangle \\ \langle \partial _2 x, \partial _1 n\rangle &{}\quad \langle \partial _2 x, \partial _2 n\rangle \end{pmatrix},&S&:={\text {I}}^{-1}{\text {II}}. \end{aligned}$$

The following observation is crucial for the definition of the mean and Gauss curvatures via the shape operator.

### Lemma 1

If the pair (*x*, *n*) is coupled, the second fundamental form ${\text {II}}$ is symmetric.

### Proof

Differentiating the equation $\langle \Delta x, n+n_1\rangle =0$ yields $\langle \Delta x, \partial n+\partial n_1\rangle =-\langle \partial \Delta x, n+n_1\rangle$. Using the assumptions $\partial x\perp n$ and $\partial x_1 \perp n_1$ completes the proof.

$$\square $$

Symmetry of the second fundamental form is equivalent to the selfadjointness of the shape operator *S* w.r.t. the inner product induced by the first fundamental form. In case of symmetry, the eigenvalues of *S* are real.

### Definition 4

Let (*x*, *n*) be a coupled semidiscrete surface and let $\kappa _1$, $\kappa _2\in \mathbb {R}$ be the eigenvalues of the shape operator *S*. Then the *mean curvature* *H* and the *Gauss curvature* *K* are defined as

$$\begin{aligned} H:=\frac{1}{2}{\text {tr}}(S)=\frac{\kappa _1+\kappa _2}{2} \quad \text {and}\quad K:=\det (S)=\frac{\det {\text {II}}}{\det {\text {I}}}=\kappa _1\kappa _2. \end{aligned}$$

Another approach toward a meaningful curvature theory for discrete or semidiscrete surfaces uses the concept of offsets and their connection to the mean and Gauss curvatures via the Steiner formula. This viewpoint has already been examined, e.g., by Bobenko et al. [6] in the purely discrete setting, and by Karpenkov and Wallner [13] in the semidiscrete case. We are going to demonstrate that the curvatures given in Definition 4 can just as well be gained via the Steiner formula.

First we note that coupled semidiscrete surfaces naturally feature offsets.

### Lemma 2

A pair of semidiscrete surfaces (*x*, *n*) is coupled if and only if for some (and hence for all) $r\in \mathbb {R}$ the offset $(x^r,n):=(x+r\,n,n)$ is coupled.

### Proof

Since $n\in \mathbb {S}^2$, we have $\langle \partial x^r, n\rangle =\langle \partial x,n\rangle$ and $\langle \Delta x^r,n+n_1\rangle =\langle \Delta x, n+n_1\rangle$, for all $r\in \mathbb {R}$. $\square$

The relation between offsets and curvatures is established by the so-called mixed area form. The following definition is motivated by the work of Hoffmann et al. [12]. Also note the similarities to the mixed area form for parallel conjugate semidiscrete surfaces previously investigated by Karpenkov and Wallner [13].

### Definition 5

For two semidiscrete surfaces (*x*, *n*), (*y*, *n*) with the same Gauss map *n* and strip normal *N*, the *mixed area form* is given by

$$\begin{aligned} A(x,y)&:=\frac{1}{2}\left( \det (\partial _1 x,\partial _2 y,N)+\det (\partial _1 y,\partial _2 x,N)\right) \\&\phantom {:}=\frac{1}{4}\left( \det (\Delta x,\partial y+\partial y_1,N)+\det (\Delta y,\partial x+\partial x_1,N)\right) . \end{aligned}$$

It turns out that, for a coupled semidiscrete surface (*x*, *n*), the mean and Gauss curvatures from Definition 4 can be expressed in terms of the mixed area forms of the parametrization *x* and its Gauss map *n* in a way completely analogous to the smooth setting. In particular, this observation shows that the curvatures given in Definition 4 coincide with those discussed by Karpenkov and Wallner [13] in the case of circular surfaces (see Definition 6).

### Lemma 3

Let (*x*, *n*) be a coupled semidiscrete surface, then

$$\begin{aligned} \mathrm{(i)}&\;\; \det {\text {I}}=A(x,x)^2,&\mathrm{(iii)}&\;\; H = -\frac{A(x,n)}{A(x,x)},\\ \mathrm{(ii)}&\;\; K = \frac{A(n,n)}{A(x,x)},&\mathrm{(iv)}&\;\; {\text {III}}-2H{\text {II}}+K{\text {I}}=0. \end{aligned}$$

### Proof

(i) We have $$\det {\text {I}}=\Vert \pi (\partial _1 x)\times \pi (\partial _2 x)\Vert ^2=\det (\pi (\partial _1 x),\pi (\partial _2 x), N)^2=\det (\partial _1 x, \partial _2 x, N)^2=A(x,x)^2$$.
(ii) Using the Binet–Cauchy identity, we compute
  $$\begin{aligned} \det {\text {II}}&=\langle \pi (\partial _1 x)\times \pi (\partial _2 x),\partial _1 n\times \partial _2 n\rangle \\&=\det (\pi (\partial _1 x),\pi (\partial _2 x),N)\Vert \partial _1 n\times \partial _2 n\Vert =A(x,x)A(n,n). \end{aligned}$$
(iii) Likewise, we obtain
  $$\begin{aligned} A(x,n)&=\frac{1}{2}\left( \det (\pi (\partial _1 x),\partial _2 n,N)+\det (\partial _1 n,\pi (\partial _2 x),N)\right) \\&=\frac{1}{2A(x,x)}\left( \Vert \pi (\partial _1 x)\Vert ^2\langle \pi (\partial _2 x), \partial _2 n\rangle {-}\langle \pi (\partial _1 x), \pi (\partial _2 x)\rangle \langle \pi (\partial _1 x),\partial _2 n\rangle \right. \\&\quad \phantom {=}{+}\left. \Vert \pi (\partial _2 x)\Vert ^2\langle \pi (\partial _1 x), \partial _1 n\rangle {-}\langle \pi (\partial _1 x), \pi (\partial _2 x)\rangle \langle \pi (\partial _2 x),\partial _1 n\rangle \right) \\&=-\frac{A(x,x)}{2}{\text {tr}}(S). \end{aligned}$$
(iv) By the Cayley–Hamilton theorem $S^2-{\text {tr}}(S)S+\det (S)E=0$, which yields the last equation. $\square$

### Corollary 1

(Semidiscrete Steiner formula) Let (*x*, *n*) be a coupled semidiscrete surface with offset $(x^r,n)=(x+r\,n,n)$, $r\in \mathbb {R}$. Then

$$\begin{aligned} A(x^r,x^r)=(1-2Hr+Kr^2)A(x,x). \end{aligned}$$

Next, we recapitulate the notion of semidiscrete isothermic parametrizations.

## Semidiscrete isothermic surfaces

A smooth parametrization is called isothermic, if it is a conformal curvature line parametrization, possibly upon a reparametrization of independent variables.

A discrete analog of curvature line parametrizations is given, for example, by circular nets, i.e., quadrilateral meshes with the property that each face possesses a circumcircle. They have been the topic of various contributions from the perspective of discrete differential geometry and integrable systems (see, e.g., [3, 4, 7, 9, 14]). Among all quadrilateral meshes, circular nets are the only ones which posses nontrivial vertex offsets, i.e., parallel meshes at constant vertexwise distance (cf. Pottmann et al. [18]). In particular, choosing an arbitrary offset direction resp. normal vector at one vertex determines the normal vectors at all other vertices.

The following semidiscrete version of circular nets was first investigated by Pottmann et al. [19]. They can be understood as semidiscrete curvature line parametrizations in exactly the same manner as their purely discrete counterparts.

### Definition 6

A semidiscrete surface (*x*, *n*) is called *circular*, if

- for each corresponding pair of points *x*, $x_1$ there is a circle $\mathcal {C}$ passing through these points and being tangent to $\partial x$, $\partial x_1$ there, and
- the Gauss map *n* is parallel to *x*, i.e., $\Delta n\parallel \Delta x$ and $\partial n\parallel \partial x$.

### Remark 1

Similar to the discrete case, a parallel Gauss map *n* of a semidiscrete surface *x* with the property (a) is completely determined by choosing *one* normal vector $n(k_0,t_0)$ arbitrarily in $\mathbb {S}^2\cap \partial x(k_0,t_0)^\perp$ (see Karpenkov and Wallner [13, Theorem 1.12]). Due to the parallelity (b), the Gauss map *n* also enjoys the property (a), and the pair (*x*, *n*) is coupled.

### Remark 2

For planar semidiscrete surfaces $x:D\rightarrow \mathbb {R}^2\cong \mathbb {C}$, circularity is equivalent to the existence of a function $s:D\rightarrow \mathbb {R}^*$, with

$$\begin{aligned} \Delta x = is\left( \frac{\partial x}{\Vert \partial x\Vert }+\frac{\partial x_1}{\Vert \partial x_1\Vert }\right) . \end{aligned}$$

We adopt the following definition of semidiscrete isothermic surfaces from Müller and Wallner [16].

### Definition 7

A circular semidiscrete surface (*x*, *n*) is called *isothermic*, if there exist positive semidiscrete functions $\nu$, $\sigma$, and $\tau$, such that

$$\begin{aligned} \Vert \Delta x\Vert ^2=\sigma \nu \nu _1, \quad \Vert \partial x\Vert ^2=\tau \nu ^2, \quad \text {and}\quad \partial \sigma =\Delta \tau =0. \end{aligned}$$

An isothermic function $g:D\rightarrow \mathbb {C}$ is called *holomorphic*.

In analogy to the smooth and purely discrete settings, for circular semidiscrete surfaces *x*, isothermicity is equivalent to the existence of a Christoffel dual (see Müller and Wallner [16, Theorem 11]). Recall that a semidiscrete surface *x* is called *conjugate*, if $\{\Delta x,\partial x, \partial x_1\}$ is linearly dependent throughout.

### Definition 8

Two conjugate semidiscrete surfaces *x*, $x^*$ are *dual* to each other, if there exists a positive semidiscrete function $\nu$, such that

$$\begin{aligned} \Delta x^*=\frac{1}{\nu \nu _1}\Delta x\quad \text {and}\quad \partial x^*=-\frac{1}{\nu ^2}\partial x. \end{aligned}$$

In this case, $x^*$ is called the *Christoffel dual* of *x*.

### Remark 3

Using the notation of Definition 7, the dual $x^*$ of a semidiscrete isothermic surface *x* fulfills

$$\begin{aligned} \Delta x^*=\frac{\sigma }{\Vert \Delta x\Vert ^2}\Delta x,\quad \partial x^*=-\frac{\tau }{\Vert \partial x\Vert ^2}\partial x,\quad \text {and}\quad A(x,x^*)=0. \end{aligned}$$

### Quaternionic description of semidiscrete isothermic surfaces

Here we provide a characterization of semidiscrete isothermic surfaces in terms of quaternions, which we will use for the study of cmc surfaces. In particular, we demonstrate that, similar to the discrete situation, a semidiscrete surface is isothermic if and only if its cross ratio allows for a specific factorization.

Consider the algebra of quaternions $\mathbb {H}$ equipped with the basis $\{\mathbf {1},\mathbf {i},\mathbf {j},\mathbf {k}\}$, where $\mathbf {i}\mathbf {j}=\mathbf {k}$, $\mathbf {j}\mathbf {k}=\mathbf {i}$, $\mathbf {k}\mathbf {i}=\mathbf {j}$. Using the standard matrix representation of $\mathbb {H}$, this basis is related to the Pauli matrices $\sigma _1$, $\sigma _2$, $\sigma _3$ via

$$\begin{aligned}&\mathbf {1}=\begin{pmatrix} 1 &{}\quad 0\\ 0 &{}\quad 1 \end{pmatrix},\ \mathbf {i}=-i\sigma _1=\begin{pmatrix} 0 &{}\quad -i\\ -i &{}\quad 0 \end{pmatrix},\nonumber \\&\quad \mathbf {j}=-i\sigma _2=\begin{pmatrix} 0 &{}\quad -1\\ 1 &{}\quad 0 \end{pmatrix},\ \mathbf {k}=-i\sigma _3=\begin{pmatrix} -i &{}\quad 0\\ 0 &{}\quad i \end{pmatrix}, \end{aligned}$$

where $i=\sqrt{-1}\in \mathbb {C}$. We embed $\mathbb {R}^3$ into $\mathbb {H}$ by

2 $$\begin{aligned}&p=(p_1,p_2,p_3)^T\!\in \mathbb {R}^3\ \leftrightarrow \ p=p_1\,\mathbf {i}+p_2\,\mathbf {j}+p_3\,\mathbf {k}\nonumber \\&\quad =\!\begin{pmatrix} -ip_3 &{} \quad -ip_1-p_2\\ -ip_1+p_2 &{} \quad ip_3 \end{pmatrix}\!\in {\text {Im}}\mathbb {H}. \end{aligned}$$

Then, the scalar product is expressed as $\langle p,q\rangle =-\frac{1}{2}{\text {tr}}(pq)$.

The identification (2) can be used to define a cross ratio of four possibly non-coplanar points in three-dimensional space up to inner automorphisms. It is known that discrete isothermic surfaces can be defined by the property that the cross ratio of each face allows for a specific factorization (cf. Bobenko and Pinkall [4]). We are going to analyze how this property translates to the semidiscrete case.

#### Definition 9

For a semidiscrete surface *x* in $\mathbb {R}^3\cong {\text {Im}}\mathbb {H}$, we define the function $Q[x]:D\rightarrow \mathbb {H}$ via

$$\begin{aligned} Q[x]:=(\partial x)(\Delta x)^{-1}(\partial x_1)(\Delta x)^{-1}, \end{aligned}$$

and call the unordered pair

$$\begin{aligned} \left\{ q[x], \bar{q}[x]\right\} :={\text {Re}}Q[x]\pm i\left\| {\text {Im}}Q[x]\right\| \end{aligned}$$

the *cross ratio* of *x*.

The cross ratio of four points in $\mathbb {R}^3$ is known to be Möbius invariant (see, e.g., Bobenko and Pinkall [4, Lemma 1]). By a limit argument this property immediately carries over to *q*[*x*], $\bar{q}[x]$. Another important feature is that the cross ratio of four points is real if and only if they lie on a circle. An analogous property holds in the semidiscrete case.

#### Lemma 4

A semidiscrete surface *x* is circular if and only if its cross ratio is real. In this case, the vectors $\partial x$, $\partial x_1$ lie to the same side of the line spanned by $\Delta x$ if and only if $Q[x]<0$.

#### Proof

At each point $(k,t)\in D$ there is a Möbius transformation $\mu$, such that , , and . Thus, by the Möbius invariance of the cross ratio, we have

$$\begin{aligned} {\text {Re}}Q[x]&={\text {Re}}\left( \mathbf {j}\mathbf {i}^{-1}\partial (\mu \circ x)_1\mathbf {i}^{-1}\right) ={\text {Re}}\left( \mathbf {i}\mathbf {j}\partial (\mu \circ x)_1\mathbf {i}^{-1}\right) \\&={\text {Re}}\left( \mathbf {j}\partial (\mu \circ x)_1\right) =-\left\langle \partial (\mu \circ x),\partial (\mu \circ x)_1\right\rangle ,\\ \Vert {\text {Im}}Q[x]\Vert&=\left\| {\text {Im}}\left( \mathbf {j}\partial (\mu \circ x)_1\right) \right\| =\left\| \partial (\mu \circ x)\times \partial (\mu \circ x)_1\right\| . \end{aligned}$$

Hence, the cross ratio is real iff $\partial (\mu \circ x)\parallel \partial (\mu \circ x)_1$, which means that the vector $\partial (\mu \circ x)_1$ anchored at $\mu (x_1)$ is tangent to the circle defined by $\mu (x)$, $\mu (x_1)$, and $\partial (\mu \circ x)$. Moreover, the cross ratio is negative iff the vectors $\partial (\mu \circ x)$ and $\partial (\mu \circ x)_1$ point to the same direction. By applying the inverse Möbius transformation $\mu ^{-1}$, the statement follows immediately. $\square$

The following lemma provides us with a characterization of semidiscrete isothermic surfaces in terms of their cross ratios.

#### Lemma 5

A semidiscrete surface *x* is isothermic if and only if there exist positive semidiscrete functions $\sigma$ and $\tau$, such that

$$\begin{aligned} Q[x]=-\frac{\tau }{\sigma }\quad \text {and}\quad \partial \sigma =\Delta \tau =0. \end{aligned}$$

In this case, $Q[x]=-\frac{\Vert \partial x\Vert \Vert \partial x_1\Vert }{\Vert \Delta x\Vert ^2}$.

#### Proof

Let *x* be an isothermic semidiscrete surface with $\nu$, $\sigma$, and $\tau$ as in Definition 7. Moreover, for each fixed $(k,t)\in D$, let the Möbius transformation $\mu$ be defined by , , and . Then, for each $\mu$, there exists $\rho >0$, such that $\Vert \mu (x)-\mu (y)\Vert ^2=\rho (x)\rho (y)\Vert x-y\Vert ^2$, for all $x,y\in \mathbb {R}^3$. This also implies that $\Vert d_x\mu (v)\Vert ^2=\rho (x)^2\Vert v\Vert ^2$, for a tangent vector *v* attached to *x*. Thus, $1=\Vert \mu (x)-\mu (x_1)\Vert ^2=\rho (x)\rho (x_1)\Vert \Delta x\Vert ^2$, and by the previous lemma we get

$$\begin{aligned} Q[x]= & {} -\langle \partial (\mu \circ x),\partial (\mu \circ x)_1\rangle =-\Vert \partial (\mu \circ x)\Vert \Vert \partial (\mu \circ x)_1\Vert \\= & {} -\frac{\Vert \partial x\Vert \Vert \partial x_1\Vert }{\Vert \Delta x\Vert ^2}=-\frac{\tau }{\sigma }. \end{aligned}$$

Conversely, assume that $Q[x]=-\frac{\tau }{\sigma }$, with $\partial \sigma =\Delta \tau =0$. By the previous lemma *x* is circular and the vectors $\partial x$, $\partial x_1$ lie to the same side of the line spanned by $\Delta x$. Hence, by the observations above, we have $\frac{\Vert \partial x\Vert \Vert \partial x_1\Vert }{\Vert \Delta x\Vert ^2}=-Q[x]=\frac{\tau }{\sigma }$. Setting $\nu :=\frac{1}{\sqrt{\tau }}\Vert \partial x\Vert$ completes the proof. $\square$

## Semidiscrete minimal surfaces

Smooth minimal surfaces in $\mathbb {R}^3$ can be defined in several equivalent ways, e.g. by locally minimizing the surface area or by having vanishing mean curvature. An isothermic minimal surface is determined by the property of being Christoffel dual to its Gauss map, giving rise to their well known Weierstrass–Enneper representation. This section is concerned with *semidiscrete* minimal surfaces, which do not fully enjoy these properties.

### Definition 10

A coupled semidiscrete surface (*x*, *n*) is called *minimal*, if its mean curvature *H* vanishes identically.

It has already been noted by Müllner and Wallner [16] that semidiscrete *isothermic* minimal surfaces are Christoffel dual to their Gauss map. Similar to the smooth case, this observation leads to a Weierstrass type representation, as demonstrated by Rossman and Yasumoto [20]. In turn, this representation gives rise to a one-parameter family of associated surfaces. These are however no longer isothermic, which has made it difficult to understand their minimality in the discrete and semidiscrete settings so far.

Let us recall the Weierstrass representation. Let $g:\mathbb {Z}\times \mathbb {R}\supseteq D\rightarrow \mathbb {C}$ be a semidiscrete holomorphic function with $\nu _g$, $\sigma _g$, and $\tau _g$ as in Definition 7. It is straightforward to show that the composition of *g* with the inverse of the stereographic projection, given by

$$\begin{aligned} n:=\frac{1}{|g|^2+1}\left( 2{\text {Re}}(g),\ 2{\text {Im}}(g),\ |g|^2-1 \right) ^T, \end{aligned}$$

is isothermic with $\nu =\frac{2\nu _g}{|g|^2+1}$, $\tau =\tau _g$, and $\sigma =\sigma _g$. Now, the Christoffel dual *x* of *n* is uniquely determined, up to translation, as solution of the system

$$\begin{aligned} \Delta x=\frac{\sigma }{\Vert \Delta n\Vert ^2}\Delta n\quad \text {and}\quad \partial x&=-\frac{\tau }{\Vert \partial n\Vert ^2}\partial n. \end{aligned}$$

We see immediately that $A(x,n)=0$, so the semidiscrete surface (*x*, *n*) is minimal. Moreover, it has been verified by Rossman and Yasumoto [20] that any semidiscrete isothermic minimal surface can be described in this way by some semidiscrete holomorphic function *g*.

As already mentioned before, the Weierstrass representation immediately gives rise to the associated family of an isothermic minimal surface.

### Definition 11

Let (*x*, *n*) be a semidiscrete isothermic minimal surface arising from a semidiscrete holomorphic function *g* with $\sigma$ and $\tau$ as in Definition 7. Then the *associated family* $(x^\alpha ,n)$, $\alpha \in \mathbb {R}$, of (*x*, *n*) is defined, up to translation, as solution of the system

$$\begin{aligned} \Delta x^\alpha&=\frac{\sigma }{2}{\text {Re}}(\lambda \phi ),\text {with } \phi :=\frac{1}{\Delta g} \left( 1-gg_1,\ i(1+gg_1),\ g+g_1 \right) ^T,\quad \text {and}\\ \partial x^\alpha&=-\frac{\tau }{2}{\text {Re}}(\lambda \psi ),\quad \text {with~} \psi :=\frac{1}{\partial g} \left( 1-g^2,\ i(1+g^2),\ 2g \right) ^{T},\quad \text {where~} \lambda :=e^{i\alpha }. \end{aligned}$$

### Lemma 6

For every semidiscrete isothermic minimal surface (*x*, *n*) the members of its associated family $(x^\alpha ,n)$ are well defined and coupled.

### Proof

To show the existence of $x^\alpha$, we check the compatibility condition $\partial \Delta x^\alpha = \Delta \partial x^\alpha$. Using the abbreviation

$$\begin{aligned}\omega :=\left( g^2\partial g_1-g_1^2\partial g-\partial \Delta g,~~ i(g_1^2\partial g-g^2\partial g_1-\partial \Delta g),~~ 2(g_1\partial g-g\partial g_1) \right) ^T, \end{aligned}$$

and the fact that $\partial \sigma =\Delta \tau =0$, one can compute

$$\begin{aligned} \partial \left( \Delta x^\alpha \right)&=\frac{\sigma }{2}{\text {Re}}\left( \frac{\lambda }{(\Delta g)^2}\ \omega \right) =\frac{\tau |\Delta g|^2}{2|\partial g||\partial g_1|}{\text {Re}}\left( \frac{\lambda }{(\Delta g)^2}\ \omega \right) \\&=\frac{\tau }{2}{\text {Re}}\left( \frac{\lambda \Delta \bar{g}}{|\partial g||\partial g_1|\Delta g}\ \omega \right) \mathop {=}\limits ^{(*)}-\frac{\tau }{2}{\text {Re}}\left( \frac{\lambda }{\partial g\partial g_1}\ \omega \right) =\Delta \left( \partial x^\alpha \right) . \end{aligned}$$

Note that the equality $(*)$ follows from the circularity of the mapping *g* (cf. Remark 2):

$$\begin{aligned} \frac{\Delta \bar{g}}{|\partial g||\partial g_1|\Delta g}=\frac{-is\left( \frac{\partial \bar{g}}{|\partial g|}+\frac{\partial \bar{g}_1}{|\partial g_1|}\right) }{|\partial g||\partial g_1|is\left( \frac{\partial g}{|\partial g|}+\frac{\partial g_1}{|\partial g_1|}\right) }=-\frac{1}{\partial g\partial g_1}. \end{aligned}$$

Finally, direct computations show $\langle \partial x^\alpha ,n\rangle =0$ and $\langle \Delta x^\alpha ,n\rangle$ $=$ $-\langle \Delta x^\alpha ,n_1\rangle$ $=$ $-\frac{\sigma }{2}{\text {Re}}(\lambda )$. This concludes the proof. $\square$

**Fig. 1:**
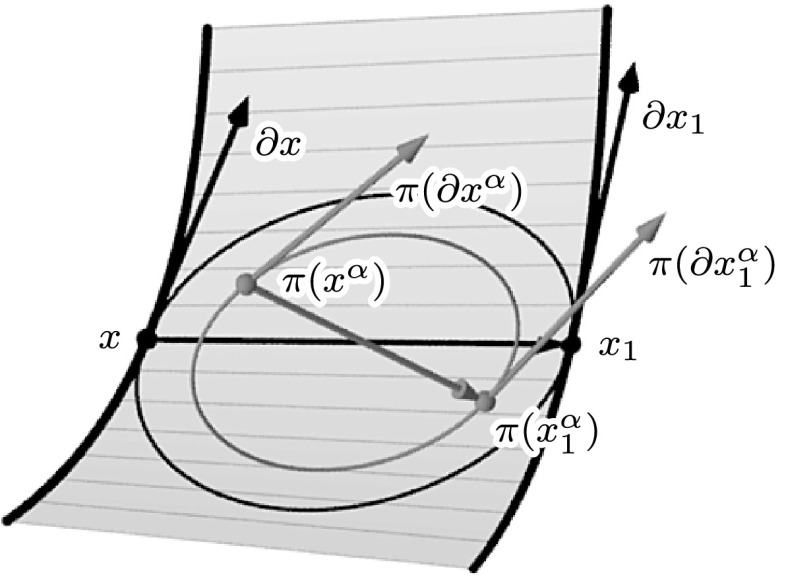
An infinitesimal quadrilateral $\{x$, $x_1$, $\partial x$, $\partial x_1\}$ (*black*) of a semidiscrete isothermic minimal surface and the corresponding projected infinitesimal quadrilateral $\{\pi (x^\alpha )$, $\pi (x_1^\alpha )$, $\pi (\partial x^\alpha )$, $\pi (\partial x_1^\alpha )\}$ (*gray*) of a member of the associated family

In order to show that the members of the associated family are indeed minimal, we follow Hoffmann et al. [12]. The key observation is as follows: Consider an (infinitesimal) quadrilateral of any member of such a family and orthogonally project it in direction of the face normal *N*. Then the resulting (infinitesimal) quadrilateral is a rotated and scaled version of the corresponding (infinitesimal) quadrilateral of the original isothermic surface (cf. Fig. 1). As a first step toward this result we provide a semidiscrete version of [12, Lemma 24].

### Lemma 7

Let $(x^\alpha ,n)$ denote the associated family of a semidiscrete isothermic minimal surface (*x*, *n*). Then, for each $\alpha \in \mathbb {R}$, we have

$$\begin{aligned} \Delta x^\alpha&=\frac{\Vert \Delta x\Vert ^2}{\sigma }\left( \cos \alpha \,\Delta n-\sin \alpha \,\Delta n\times n \right) ,\quad \text {and}\\ \partial x^\alpha&=-\frac{\Vert \partial x\Vert ^2}{\tau } \left( \cos \alpha \,\partial n-\sin \alpha \,\partial n\times n \right) . \end{aligned}$$

### Proof

Lemma 6 implies $\Delta x^\alpha \perp (n+n_1)$ and $\partial x^\alpha \perp n$. Hence, $\Delta x^\alpha$ is a linear combination of $\Delta n$ and $\Delta n\times n$, whereas $\partial x^\alpha$ is a linear combination of $\partial n$ and $\partial n\times n$. Moreover,

$$\begin{aligned} \Delta x^\alpha&=\frac{\sigma }{2}{\text {Re}}(\lambda \phi )=\frac{\sigma }{2}\left( \cos \alpha \,{\text {Re}}(\phi )-\sin \alpha \,{\text {Im}}(\phi )\right) ,\quad \text {and}\\ \partial x^\alpha&=-\frac{\tau }{2}{\text {Re}}(\lambda \psi )=-\frac{\tau }{2} \left( \cos \alpha \,{\text {Re}}(\psi )-\sin \alpha \,{\text {Im}}(\psi )\right) . \end{aligned}$$

Since by construction the surfaces (*x*, *n*) and (*n*, *n*) are dual to each other, we know that ${\text {Re}}(\phi )=\frac{2}{\sigma \nu \nu _1}\Delta n=\frac{2}{\Vert \Delta n\Vert ^2}\Delta n=\frac{2\Vert \Delta x\Vert ^2}{\sigma ^2}\Delta n$ and ${\text {Re}}(\psi )=\frac{2}{\tau \nu ^2}\partial n=\frac{2}{\Vert \partial n\Vert ^2}\partial n=\frac{2\Vert \partial x\Vert ^2}{\tau ^2}\partial n$.

It remains to show that ${\text {Im}}(\phi )=\frac{2\Vert \Delta x\Vert ^2}{\sigma ^2}(\Delta n\times n)$ and ${\text {Im}}(\psi )=\frac{2\Vert \partial x\Vert ^2}{\tau ^2}(\partial n\times n)$. Firstly, it is easy to verify that $\langle \phi ,\bar{\phi }\rangle _{\mathbb {C}^3}=1$ and $\langle \psi ,\bar{\psi }\rangle _{\mathbb {C}^3}=0$, which implies that ${\text {Im}}(\phi )$ and ${\text {Im}}(\psi )$ are perpendicular to ${\text {Re}}(\phi )$ and ${\text {Re}}(\psi )$, respectively. Furthermore, we check that $\langle {\text {Im}}(\phi ),n\rangle =0$ and $\langle {\text {Im}}(\psi ),n\rangle =0$, so ${\text {Im}}(\phi )\parallel \Delta n\times n$ and ${\text {Im}}(\psi )\parallel \partial n\times n$. Finally, we compute

$$\begin{aligned} \langle {\text {Im}}(\phi ),\Delta n\times n\rangle&=\det ({\text {Im}}(\phi ),\Delta n,n)={\text {Im}}\left( \det (\phi ,n_1,n) \right) \\&=\frac{2(|g|^2|g_1|^2+g_1\bar{g}+g\bar{g}_1+1)}{(1+|g|^2)(1+|g_1|^2)}=2-\frac{2|\Delta g|^2}{(1+|g|^2)(1+|g_1|^2)}\\&=2-\frac{\Vert \Delta n\Vert ^2}{2}=\frac{\Vert n_1+n\Vert ^2}{2}=\frac{2\Vert \Delta n\times n\Vert ^2}{\Vert \Delta n\Vert ^2}\\&=\frac{2\Vert \Delta x\Vert ^2}{\sigma ^2}\Vert \Delta n\times n\Vert ^2,\\ \langle {\text {Im}}(\psi ),\partial n\times n\rangle&=\det ({\text {Im}}(\psi ),\partial n,n)=\frac{2|\partial g|^2}{(1+|g|^2)^2}\det ({\text {Im}}(\psi ),{\text {Re}}(\psi ),n)\\&=\frac{|\partial g|^2}{(1+|g|^2)^2}{\text {Im}}\left( \det (\psi ,\bar{\psi },n) \right) =2, \end{aligned}$$

where we have used the fact that *n* maps to $\mathbb {S}^2$. Thus, we have ${\text {Im}}(\phi )=\frac{2\Vert \Delta x\Vert ^2}{\sigma ^2}(\Delta n\times n)$ and ${\text {Im}}(\psi )=\frac{2}{\Vert \partial n\times n\Vert ^2}(\partial n\times n)=\frac{2}{\Vert \partial n\Vert ^2}(\partial n\times n)=\frac{2\Vert \partial x\Vert ^2}{\tau ^2}(\partial n\times n)$. This concludes the proof. $\square$

The rotation property mentioned above is stated as follows:

### Lemma 8

Let $(x^\alpha ,n)$ be the associated family of a semidiscrete isothermic minimal surface (*x*, *n*) and let $\pi$ denote the orthogonal projection in direction of the strip normal *N*. Then, for all $\alpha$, the infinitesimal quadrilateral $\{\pi (x^\alpha ),\pi (x_1^\alpha ),\pi (\partial x^\alpha ),\pi (\partial x_1^\alpha )\}$ is a rotated and scaled version of the infinitesimal quadrilateral $\{x,x_1,\partial x,\partial x_1\}$ (cf. Fig. 1).

### Proof

By the previous lemma,

$$\begin{aligned} \pi (\Delta x^\alpha )&=\frac{\Vert \Delta x\Vert ^2}{\sigma }\left( \cos \alpha \,\Delta n-\sin \alpha \,\pi (\Delta n\times n) \right) ,\quad \text {and}\\ \pi (\partial x^\alpha )&=-\frac{\Vert \partial x\Vert ^2}{\tau } \left( \cos \alpha \,\partial n-\sin \alpha \,\pi (\partial n\times n) \right) . \end{aligned}$$

The orthogonality $\pi (\Delta n\times n)\perp \Delta n$ implies

$$\begin{aligned} \Vert \pi (\Delta x^\alpha )\Vert ^2&=\frac{\Vert \Delta x\Vert ^4}{\sigma ^2}\left( \cos (\alpha )^2\Vert \Delta n\Vert ^2-\sin (\alpha )^2\Vert \pi (\Delta n\times n)\Vert ^2 \right) \\&=\frac{\Vert \Delta x\Vert ^4}{\sigma ^2} \left( \cos (\alpha )^2\Vert \Delta n\Vert ^2-\sin (\alpha )^2\cos (\mu )^2\Vert (\Delta n\times n)\Vert ^2 \right) \\&=\Vert \Delta x\Vert ^2\left( \cos (\alpha )^2-\sin (\alpha )^2\cos (\mu )^2 \left\| \frac{n+n_1}{2}\right\| ^2\right) \\&=\Vert \Delta x\Vert ^2 \left( \cos (\alpha )^2-\sin (\alpha )^2d^2 \right) , \end{aligned}$$

where $\mu :=\angle (\Delta n\times n,\pi (\Delta n\times n))=\angle (\frac{n+n_1}{2},N)$, and *d* denotes the distance between the origin and the center of the circle $\mathcal {C}$ determined by $\{n,n_1,\partial n,\partial n_1\}$ in the same manner as in Definition 6 (a). Likewise, $\pi (\partial n\times n)\perp \partial n$ implies

$$\begin{aligned} \Vert \pi (\partial x^\alpha )\Vert ^2&=\frac{\Vert \partial x\Vert ^4}{\tau ^2} \left( \cos (\alpha )^2\Vert \partial n\Vert ^2-\sin (\alpha )^2\Vert \pi (\partial n\times n)\Vert ^2 \right) \\&=\frac{\Vert \partial x\Vert ^4}{\tau ^2} \left( \cos (\alpha )^2\Vert \partial n\Vert ^2-\sin (\alpha )^2\cos (\xi )^2\Vert \partial n\times n\Vert ^2 \right) \\&=\Vert \partial x\Vert ^2 \left( \cos (\alpha )^2-\sin (\alpha )^2\cos (\xi )^2\Vert n\Vert ^2 \right) \\&=\Vert \partial x\Vert ^2 \left( \cos (\alpha )^2-\sin (\alpha )^2d^2 \right) , \end{aligned}$$

where $\xi :=\angle (\partial n\times n,\pi (\partial n\times n))=\angle (n,N)$. Analogously, we obtain

$$\begin{aligned} \Vert \pi (\partial x_1^\alpha )\Vert ^2=\Vert \partial x_1\Vert ^2 \left( \cos (\alpha )^2-\sin (\alpha )^2d^2 \right) . \end{aligned}$$

Finally, we observe that

$$\begin{aligned} \frac{\langle \pi (\Delta x^\alpha ),\Delta x\rangle }{\Vert \pi (\Delta x^\alpha )\Vert \Vert \Delta x\Vert }\,{=}\,\frac{\langle \pi (\partial x^\alpha ),\partial x\rangle }{\Vert \pi (\partial x^\alpha )\Vert \Vert \partial x\Vert }\,{=}\,\frac{\langle \pi (\partial x_1^\alpha ),\partial x_1\rangle }{\Vert \pi (\partial x_1^\alpha )\Vert \Vert \partial x_1\Vert }\,{=}\,\frac{\cos \alpha }{\sqrt{\cos (\alpha )^2{-}\sin (\alpha )^2d^2}}. \end{aligned}$$

Thus, the infinitesimal quadrilateral $\{\pi (x^\alpha ),\,\pi (x_1^\alpha ),\,\pi (\partial x^\alpha ),\,\pi (\partial x_1^\alpha )\}$ arises from the infinitesimal quadrilateral $\{x,x_1,\partial x,\partial x_1\}$ by scaling with factor $\rho _\alpha$ and rotating by the angle $\theta _\alpha$, with

$$\begin{aligned} \displaystyle \rho _\alpha =\sqrt{\cos (\alpha )^2-\sin (\alpha )^2d^2}\quad \text {and}\quad \cos \theta _\alpha =\frac{\cos \alpha }{\rho _\alpha }. \end{aligned}$$

$$\square $$

We are now able to prove the main result of the present section.

### Theorem 1

Every member $(x^\alpha ,n)$ of the associated family of a semidiscrete isothermic minimal surface (*x*, *n*) is minimal, i.e., has vanishing mean curvature.

### Proof

Recall that the rotation by an angle $\theta$ about the axis in direction of *N* can be written as

$$\begin{aligned} R_{N,\theta }(x)=\langle N,x\rangle N+\cos \theta \,(N\times x)\times N+\sin \theta \, N\times x. \end{aligned}$$

According to Lemma 8, we thus have

$$\begin{aligned} \pi (\Delta x^\alpha )&=\rho _\alpha R_{N,\theta _\alpha }(\Delta x)=\rho _\alpha \left( \cos \theta _\alpha \,\Delta x+\sin \theta _\alpha \, N\times \Delta x\right) ,\quad \text {and}\\ \pi (\partial x^\alpha +\partial x_1^\alpha )&=\rho _\alpha R_{N,\theta _\alpha }(\partial x+\partial x_1)\\&=\rho _\alpha \left( \cos \theta _\alpha \,(\partial x+\partial x_1)+\sin \theta _\alpha \,N\times (\partial x+\partial x_1) \right) . \end{aligned}$$

Since

$$\begin{aligned} N\times \Delta x&=\frac{1}{\Vert \Delta n\times (\partial n+\partial n_1)\Vert }\left( \langle \Delta x,\Delta n\rangle (\partial n+\partial n_1)\right. \\&\quad \left. -\langle \Delta x,\partial n+\partial n_1\rangle \Delta n\right) ,\\ N\times (\partial x+\partial x_1)&=\frac{1}{\Vert \Delta n\times (\partial n+\partial n_1)\Vert }\left( \langle \Delta n,\partial x+\partial x_1\rangle (\partial n+\partial n_1)\right. \\&\quad \left. -\langle \partial n+\partial n_1,\partial x+\partial x_1\rangle \Delta n \right) , \end{aligned}$$

the term $4A(x^\alpha ,n)=\det (\pi (\Delta x^\alpha ),\partial n+\partial n_1,N )+\det (\Delta n,\pi (\partial x^\alpha +\partial x_1^\alpha ),N )$ vanishes for all $\alpha \in \mathbb {R}$ if and only if $A(x,n)=0$ and $\langle \Delta x,\partial n+\partial n_1\rangle =\langle \Delta n,\partial x+\partial x_1\rangle$. Both equations hold since (*x*, *n*) is an isothermic minimal surface (cf. Remark 3 and Lemma 1). $\square$

In the smooth setting, the Gauss curvature of the members of the associated family of a minimal surface is independent of the parameter $\alpha$ as well. This is no longer the case in the discrete and semidiscrete situations.

### Lemma 9

Under the assumptions of Theorem 1, the Gauss curvature $K^\alpha$ of $(x^\alpha ,n)$ obeys

$$\begin{aligned} K^\alpha =\frac{K^0}{\cos (\alpha )^2+\sin (\alpha )^2d^2}, \end{aligned}$$

where *d* is the distance between the origin and the center of the circle $\mathcal {C}$ determined by $\{n,n_1,\partial n,\partial n_1\}$.

### Proof

From the proof of Lemma 8 it follows that $A(x^\alpha ,x^\alpha )=\rho _\alpha ^2A(x,x)$, with $\rho _\alpha ^2=\cos (\alpha )^2+\sin (\alpha )^2d^2$. $\square$

We conclude this section by proving that the *conjugate surface* $(x^\frac{\pi }{2},n)$ of a semidiscrete isothermic minimal surface (*x*, *n*) is an asymptotic parametrization, in analogy to the smooth and discrete cases. Semidiscrete asymptotic parametrizations have been studied, e.g., by Wallner [23]. Here, the notation $x_{\bar{1}}$ indicates an index shift in the opposite direction: $x_{\bar{1}}(k,t):=x(k-1,t)$.

### Lemma 10

Let (*x*, *n*) be a semidiscrete isothermic minimal surface with associated family $(x^\alpha ,n)$. Then the conjugate surface $(x^\frac{\pi }{2},n)$ is an asymptotic parametrization, i.e., the vectors

$$\begin{aligned} \partial x^\frac{\pi }{2},\ \partial ^2x^\frac{\pi }{2},\ \Delta x^\frac{\pi }{2}=x^\frac{\pi }{2}_1-x^\frac{\pi }{2},\quad \text {and}\quad \Delta x^\frac{\pi }{2}_{\bar{1}}=x^\frac{\pi }{2}-x^\frac{\pi }{2}_{\bar{1}} \end{aligned}$$

lie in a plane with unit normal vector *n*.

### Proof

From Lemma 7, we have

$$\begin{aligned} \Delta x^{\frac{\pi }{2}}=-\frac{\Vert \Delta x\Vert ^2}{\sigma }\Delta n\times n\quad \text {and}\quad \partial x^{\frac{\pi }{2}}=\frac{\Vert \partial x\Vert ^2}{\tau }\partial n\times n. \end{aligned}$$

The computation $\partial ^2x^\frac{\pi }{2}=\partial (\frac{\Vert \partial x\Vert ^2}{\tau })\partial n\times n+\frac{\Vert \partial x\Vert ^2}{\tau }(\partial ^2 n)\times n$ concludes the proof. $\square$

### Example 1

**Fig. 2:**
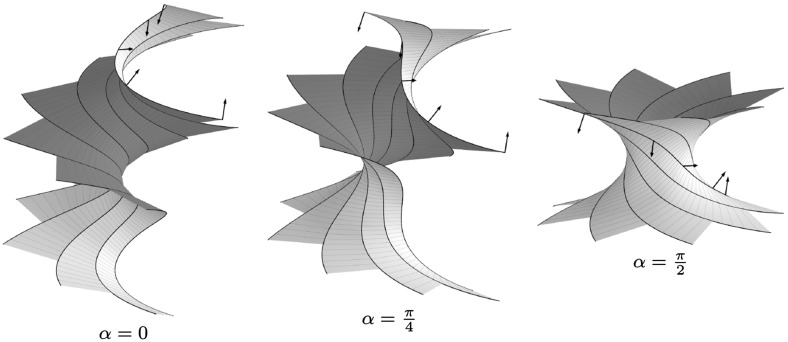
A semidiscrete helicoid (*left*) and two members of its associated family. The corresponding semidiscrete holomorphic function *g* described in Example 1 takes $r=\sqrt{2}$, $\beta =\pi /4$, and $\varphi =\pi /8$ as parameters. For $\alpha =\pi /2$ we obtain a semidiscrete asymptotic parametrization of a catenoid (*right*)

As an example we investigate the associated family of a semidiscrete helicoid (cf. Fig. 2). In classical differential geometry, an isothermic parametrization of the helicoid is gained from the Weierstrass data $f(z)=1/(1+i)$ and $g(z)=\exp ((1+i)z)$. Its conjugate is an asymptotically parametrized catenoid. A semidiscrete analog of the holomorphic map $z\mapsto \exp (az)$, $a\in \mathbb {C}$, has been proposed by Müller [15, Theorem 7] and is given by

$$\begin{aligned} g(k,t)=\exp \left( r\exp (i\beta )t+(i\varphi +\log \mu )k \right) , \end{aligned}$$

with $r\in \mathbb {R}_+$, $\beta \in \mathbb {R}$, and $\varphi \in \mathbb {R}^*$, such that $\mu :=\frac{\cos (\beta +\varphi /2)}{\cos (\beta -\varphi /2)}>0$. It is straightforward to check that *g* is holomorphic with

$$\begin{aligned} \nu _g=\mu ^k\exp (r\cos (\beta )t),\quad \sigma _g=\frac{2\sin (\varphi )^2}{\cos (2\beta )+\cos (\varphi )},\quad \text {and}\quad \tau _g=r^2. \end{aligned}$$

## Semidiscrete cmc surfaces

This section focuses on semidiscrete cmc surfaces, which enjoy nonzero constant mean curvature. In contrast to minimal surfaces, isothermic cmc surfaces are characterized by having a Christoffel dual at constant distance. This observation immediately follows from the fact that cmc surfaces are linear Weingarten surfaces.

### Lemma 11

Let (*x*, *n*) be coupled. Then the mean and Gauss curvatures of the offsets $(x^r,n)=(x+r\,n,n)$, $r\in \mathbb {R}$, are given by

$$\begin{aligned} H^r=\frac{H-Kr}{1-2Hr+Kr^2}\quad \text {and}\quad K^r=\frac{K}{1-2Hr+Kr^2}. \end{aligned}$$

If $H={\text {const.}}\ne 0$, $(x^r,n)$ is a linear Weingarten surface, i.e., there exist $a, b \in \mathbb {R}$ only depending on *r* and *H*, such that $aH^r+bK^r=1$. An analogous result applies to constant Gauss curvature surfaces.

### Proof

In case $H={\text {const.}}\ne 0$, we set $a:=\frac{1}{H}-2r$ and $b:=\frac{r}{H}-r^2$. If $K={\text {const.}}\ne 0$, we set $a:=-2r$ and $b:=\frac{1}{K}-r^2$. $\square$

### Corollary 2

If the surface (*x*, *n*) has constant mean curvature $H=\frac{1}{h}$, then the offset $(x^h,n)=(x+h\,n, n)$ has constant mean curvature $H^h=-H$, and the central surface $(x^\frac{h}{2},n)=(x+\frac{h}{2}\,n,n)$ has constant positive Gauss curvature $K^\frac{h}{2}=4H^2$.

### Corollary 3

For a coupled semidiscrete surface (*x*, *n*) and its offset $(\hat{x},n):=(x+n,n)$, we have the equivalence

$$\begin{aligned} A(x,\hat{x})=0\iff H=-\frac{A(x,n)}{A(x,x)}=1\iff \hat{H}=-\frac{A(\hat{x},n)}{A(\hat{x},\hat{x})}=-1. \end{aligned}$$

We dedicate the rest of this paper to the description of semidiscrete isothermic cmc surfaces in terms of a pair of linear first-order matrix partial differential equations called a Lax pair. Similar to the case of minimal surfaces, this representation directly includes the definition of a one-parameter family of associated surfaces. We consider only the case $H=\pm 1$, since it can always be achieved by scaling.

### The Lax pair representation of smooth cmc surfaces

We briefly recapitulate the smooth situation. For details see Bobenko [2] or Fujimori et al. [10]. Consider a smooth conformal immersion

$$\begin{aligned} x:\mathbb {C}\supseteq D\rightarrow \mathbb {R}^3:z\mapsto x(z), \end{aligned}$$

with complex coordinate $z=s+it$. Conformality means that

$$\begin{aligned} \langle \partial _z x, \partial _z x\rangle =\langle \partial _{\bar{z}} x, \partial _{\bar{z}} x\rangle =0 \end{aligned}$$

throughout the parameter domain, where $\langle \cdot ,\cdot \rangle$ denotes the bilinear complex extension of the standard Euclidean inner product and $\partial _z$, $\partial _{\bar{z}}$ are the Wirtinger derivatives $\partial _z=\frac{1}{2}(\partial _s-i\partial _t)$ and $\partial _{\bar{z}}=\frac{1}{2}(\partial _s+i\partial _t)$.

As initiated in Sect. 3.1, we identify $\mathbb {R}^3$ with the set of purely imaginary quaternions ${\text {Im}}\mathbb {H}$. Thereby, rotating a point $x\in \mathbb {R}^3$ translates to the conjugation of $x\in {\text {Im}}\mathbb {H}$ by a unit quaternion *q*. In the matrix representation of $\mathbb {H}$, the set of unit quaternions $\{q\in \mathbb {H}:\Vert q\Vert =1\}$ coincides with the Lie group $\mathrm {SU}_2=\{A\in \mathbb {C}^{2\times 2}:A^H=A^{-1},\ \det (A)=1\}$. The corresponding Lie algebra is $\mathfrak {su}_2=\{A\in \mathbb {C}^{2\times 2}:A^H=-A,\ {\text {tr}}(A)=0\}$. In this manner, $\mathrm {SU}_2$ is a double covering of $\mathrm {SO}_3$, which we identify with the set of positively oriented orthonormal frames.

Now, let $\Psi =\Psi (z)\in \mathrm {SU}_2$ represent the frame $(\frac{\partial _s x}{\Vert \partial _s x\Vert }, \frac{\partial _t x}{\Vert \partial _t x\Vert }, n)^T\in \mathrm {SO}_3$, where $n=\frac{\partial _s x\times \partial _t x}{\Vert \partial _s x\times \partial _t x\Vert }$. Then,

3 $$\begin{aligned} \partial _s x=e^{u/2}\Psi ^{-1}\mathbf {i}\Psi ,\quad \partial _t x=e^{u/2}\Psi ^{-1}\mathbf {j}\Psi , \quad \text {and}\quad n=\Psi ^{-1}\mathbf {k}\Psi , \end{aligned}$$

with $e^{u}=\Vert \partial _s x\Vert ^2=\Vert \partial _t x\Vert ^2$. It turns out that the frame $\Psi$ moves according to

4 $$\begin{aligned} \partial _z \Psi = \begin{pmatrix} \frac{\partial _z u}{4} &{}\quad -Qe^{-u/2}\\ \frac{1}{2}He^{u/2} &{}\quad -\frac{\partial _z u}{4} \end{pmatrix}\Psi , \qquad \partial _{\bar{z}} \Psi = \begin{pmatrix} -\frac{\partial _{\bar{z}} u}{4} &{}\quad -\frac{1}{2}He^{u/2} \\ \bar{Q}e^{-u/2} &{}\quad \frac{\partial _{\bar{z}} u}{4} \end{pmatrix}\Psi , \end{aligned}$$

where the so-called Hopf differential *Q* and the mean curvature *H* satisfy $Q=\langle \partial _z\partial _z x, n\rangle$ and $\frac{1}{2}He^u=\langle \partial _z\partial _{\bar{z}}x, n\rangle$. The integrability condition of this system, i.e., $\partial _z(\partial _{\bar{z}}\Psi )=\partial _{\bar{z}}(\partial _z\Psi )$, is equivalent to

5 $$\begin{aligned} \partial _z\partial _{\bar{z}} u= 2Q\bar{Q}e^{-u} - \frac{1}{2}H^2e^u \quad \text {and}\quad \partial _{\bar{z}}Q=\frac{1}{2}e^u\partial _z H. \end{aligned}$$

Thus, if we assume constant mean curvature, the Hopf differential is holomorphic. If in addition the surface has no umbilic points, then $Q\ne 0$ and we can achieve that $Q=\text {const.}\ne 0$ by a holomorphic change of coordinates. Moreover, Eq. (5) then are invariant with respect to the transformation $Q\mapsto \Lambda Q$, with $\Lambda =e^{2i\alpha }$, $\alpha \in \mathbb {R}$. In particular, we may assume that the Hopf differential is real, in which case *x* is isothermic. By integrating Eqs. (4) and (3) with *Q* replaced by $\Lambda Q$, we obtain a one-parameter family of surfaces $x^\alpha$ with the same constant mean curvature.

Remarkably, the solution $x^\alpha$ of the system (3) can be obtained without integration, by a formula first suggested by Sym [21] for K-surfaces and later translated by Bobenko [1, 2] to numerous other cases, including cmc surfaces in various space forms. Indeed, for any solution $\Psi =\Psi (z,\alpha )$ of the system (4) with *Q* replaced by $\Lambda Q$, the parametrization

$$\begin{aligned} x^\alpha :=-\frac{1}{H}\Psi ^{-1}\frac{\partial }{\partial \alpha }\Psi +\Psi ^{-1}\mathbf {k}\Psi , \end{aligned}$$

describes a cmc surface with metric $e^u$, mean curvature *H*, and Hopf differential $\Lambda Q$ (see [2, Thm. 5]).

For the sake of simplicity, we henceforth assume without loss of generality that $H=1$ and $Q=1/2$. Furthermore, we introduce the gauge equivalent frame

$$\begin{aligned} \widetilde{\Psi }:=\begin{pmatrix} e^{-i\alpha /2} &{}\quad 0 \\ 0 &{}\quad e^{i\alpha /2} \end{pmatrix}\Psi =\begin{pmatrix} \frac{1}{\sqrt{\lambda }} &{}\quad 0 \\ 0 &{}\quad \sqrt{\lambda } \end{pmatrix}\Psi ,\quad \text {with}\quad \lambda :=\sqrt{\Lambda }=e^{i\alpha }. \end{aligned}$$

Using the relations $\partial _s = \partial _z+\partial _{\bar{z}}$ and $\partial _t=i(\partial _z-\partial _{\bar{z}})$, the frame equations (4) with $H=1$ and $Q=\Lambda /2$ translate to

$$\begin{aligned} \partial _s\widetilde{\Psi }&=\mathcal {U}\widetilde{\Psi },\quad \text {with}\quad \mathcal {U}=\frac{1}{2} \begin{pmatrix} -i\frac{\partial _t u}{2} &{} -\frac{e^{u/2}}{\lambda }-\frac{\lambda }{e^{u/2}} \\ \lambda e^{u/2}+\frac{1}{\lambda e^{u/2}} &{} i\frac{\partial _t u}{2} \end{pmatrix},\quad \text {and}\\ \partial _t\widetilde{\Psi }&=\mathcal {V}\widetilde{\Psi },\quad \text {with}\quad \mathcal {V}=\frac{1}{2} \begin{pmatrix} i\frac{\partial _s u}{2} &{}\quad -\frac{i\lambda }{e^{u/2}}+\frac{ie^{u/2}}{\lambda } \\ i\lambda e^{u/2}-\frac{i}{\lambda e^{u/2}} &{} \quad -i\frac{\partial _s u}{2} \end{pmatrix}. \end{aligned}$$

Now, the integrability condition $\partial _s(\partial _t\widetilde{\Psi })=\partial _t(\partial _s\widetilde{\Psi })\mathrel {\Leftrightarrow }\partial _s \mathcal {V}+\mathcal {V}\mathcal {U}=\partial _t \mathcal {U}+\mathcal {U}\mathcal {V}$ is equivalent to the elliptic $\sinh$-Gordon equation:

$$\begin{aligned} \partial _{ss}u+\partial _{tt}u=-4\sinh (u). \end{aligned}$$

Finally, we note that the matrices $\mathcal {U}$ and $\mathcal {V}$ belong to the loop algebra

$$\begin{aligned} \Lambda \mathfrak {su}_2:=\{A:\mathbb {S}^1\rightarrow \mathfrak {su}_2:A(-\lambda )=\sigma _3 A(\lambda )\sigma _3\}, \end{aligned}$$

and accordingly $\widetilde{\Psi }$ lies in the corresponding loop group

$$\begin{aligned} \Lambda \mathrm {SU}_2:=\{A:\mathbb {S}^1\rightarrow \mathrm {SU}_2:A(-\lambda )=\sigma _3 A(\lambda )\sigma _3\}. \end{aligned}$$

The condition $A(-\lambda )=\sigma _3 A(\lambda )\sigma _3$ states that the elements of $\Lambda \mathrm {SU}_2$ and $\Lambda \mathfrak {su}_2$ have even functions of $\lambda$ on their diagonals and odd functions of $\lambda$ on their off-diagonals.

### A Lax pair representation of semidiscrete cmc surfaces

As demonstrated by Bobenko and Pinkall [5], the observations above can be utilized to derive a Lax pair representation of *discrete* isothermic cmc surfaces along with their associated families. However, only recently it has been verified by Hoffmann et al. [12] that the members of these associated families, which are no longer isothermic, indeed have the same constant mean curvature. In this subsection we explore similar results for *semidiscrete* surfaces.

Mimicking the smooth and discrete cases, we seek a solution $\Phi (k,t,\alpha )\in \Lambda \mathrm {SU}_2$ of the system

6 $$\begin{aligned} \Phi _1=U\Phi ,\quad \partial \Phi =V\Phi ,\quad \Phi (0,0,\alpha )=\mathbf {1}, \end{aligned}$$

with the Lax matrices

7 $$\begin{aligned} U:=\frac{1}{\gamma }\begin{pmatrix} a &{}\quad \frac{i}{u\lambda }-iu\lambda \\ \frac{i\lambda }{u}-\frac{iu}{\lambda } &{}\quad \bar{a} \end{pmatrix}\in \Lambda \mathrm {SU}_2,\quad V:=\frac{1}{\delta }\begin{pmatrix} ib &{}\quad \frac{1}{v\lambda }+v\lambda \\ -\frac{\lambda }{v}-\frac{v}{\lambda } &{} \quad -ib \end{pmatrix}\in \Lambda \mathfrak {su}_2, \end{aligned}$$

where $\lambda :=e^{i\alpha }$, $\alpha \in \mathbb {R}$, $a:\mathbb {Z}\times \mathbb {R}\rightarrow \mathbb {C}$, $b,\delta :\mathbb {Z}\times \mathbb {R}\rightarrow \mathbb {R}$, $u,v:\mathbb {Z}\times \mathbb {R}\rightarrow \mathbb {R}_+$, and $\gamma ^2:=|a|^2+u^2+u^{-2}-\lambda ^2-\lambda ^{-2}$, such that $\det (U)=1$.

The compatibility condition $\partial (\Delta \Phi )=\Delta (\partial \Phi )$ of the system (6) is equivalent to

8 $$\begin{aligned} \partial U + UV = V_1U, \end{aligned}$$

which expands to

9 $$\begin{aligned}&\partial \gamma = \Delta \delta = 0,\quad u^2 = vv_1,\nonumber \\&i\delta \partial u + (b_1 + b)u = av - \bar{a}v_1,\quad \text {and} \nonumber \\&i\delta \partial a + (b_1 - b)a = uv + uv_1 - \frac{1}{uv} - \frac{1}{uv_1}. \end{aligned}$$

To resolve the relation $u^2=vv_1$, we introduce a function $w:\mathbb {Z}\times \mathbb {R}\rightarrow \mathbb {R}$ and set $v=e^{2w}$ and $u=e^{w+w_1}$. Then, taking the real resp. imaginary parts of the Eq. (9) leads to ${\text {Im}}(a)=\frac{\delta (\partial w+\partial w_1)}{2\cosh (w-w_1)}$, $b_1=2{\text {Re}}(a)\sinh (w-w_1)-b$, $\partial {\text {Re}}(a)=-\frac{{\text {Im}}(a)}{\delta }(b_1-b)$, and

$$\begin{aligned} -\delta \partial {\text {Im}}(a)+(b_1-b){\text {Re}}(a)=2\sinh (3w+w_1)+2\sinh (w+3w_1), \end{aligned}$$

which is a semidiscrete version of the elliptic $\sinh$-Gordon equation. The analogy to the smooth case is not obvious at first glance. For a purely discrete version of this equation we refer to Pedit and Wu [17].

As in the smooth and discrete cases, we use the Sym–Bobenko formula to gain a parametrization of the semidiscrete surface related to the frame $\Phi$. In particular, we are going to investigate the following three parallel surfaces.

#### Definition 12

Let $\Phi (k,t,\alpha )\in \Lambda \mathrm {SU}_2$, $\alpha \in \mathbb {R}$, be a solution of the system $\Phi _1=U\Phi$, $\partial \Phi =V\Phi$, $\Phi (0,0,\alpha ) = \mathbf {1}$, where $U\in \Lambda \mathrm {SU}_2$ and $V\in \Lambda \mathfrak {su}_2$ are Lax matrices of the form (7) satisfying the compatibility condition (8). Then we define the following families of parallel surfaces

$$\begin{aligned} \check{x}^\alpha :=-\Phi ^{-1}\frac{\partial }{\partial \alpha }\Phi -\frac{1}{2}n^\alpha ,\quad x^\alpha :=-\Phi ^{-1}\frac{\partial }{\partial \alpha }\Phi ,\quad \hat{x}^\alpha :=-\Phi ^{-1}\frac{\partial }{\partial \alpha }\Phi +\frac{1}{2}n^\alpha , \end{aligned}$$

together with their common Gauss map $n^\alpha :=\Phi ^{-1}\mathbf {k}\Phi$.

We will see later (cf. Corollary 6) that, for $\alpha =0$, the surfaces $(\check{x}^0,n^0)$ and $(\hat{x}^0,n^0)$ constructed as in Definition 12 are Christoffel dual isothermic cmc surfaces. Consequently, the families $(\check{x}^\alpha ,n^\alpha )$ and $(\hat{x}^\alpha ,n^\alpha )$ represent their *associated families*.

At first we show that the pairs $(\check{x}^\alpha ,n^\alpha )$, $(x^\alpha , n^\alpha )$, and $(\hat{x}^\alpha , n^\alpha )$ from Definition 12 are coupled. In fact, we prove the following slightly more general result.

#### Lemma 12

Let $\Phi (k,t,\alpha )\in \mathrm {SU}_2$ be a moving frame defined by $\Phi _1=U\Phi$, $\partial \Phi =V\Phi$, $\Phi (0,0,\alpha )=\mathbf {1}$, where $U\in \mathrm {SU}_2$ and $V\in \mathfrak {su}_2$ satisfy the compatibility condition (8). Moreover, let $p,q\in \mathbb {R}$ be arbitrary coefficients. Then the semidiscrete surface $(x^\alpha ,n^\alpha )$ defined by the formula

$$\begin{aligned} x^\alpha :=p\Phi ^{-1}\frac{\partial }{\partial \alpha }\Phi +qn^\alpha ,\qquad n^\alpha :=\Phi ^{-1}\mathbf {k}\Phi , \end{aligned}$$

fulfills the constraint (1) if and only if *U* satisfies $U_{22}\frac{\partial }{\partial \alpha }U_{11}=U_{11}\frac{\partial }{\partial \alpha }U_{22}$, and *V* satisfies ${\text {tr}}(\frac{\partial }{\partial \alpha }V\mathbf {k})=0$, i.e., $\frac{\partial }{\partial \alpha }V_{11}=\frac{\partial }{\partial \alpha }V_{22}$.

#### Proof

The condition $\Delta x^\alpha \perp (n_1^\alpha +n^\alpha )$ holds iff ${\text {tr}}((x_1^\alpha -x^\alpha )(n^\alpha +n_1^\alpha ))=0$. Thus, we compute

$$\begin{aligned} (x_1^\alpha -x^\alpha )n^\alpha&=\Phi ^{-1}\left( pU^{-1}\frac{\partial }{\partial \alpha }U+qU^{-1}\mathbf {k}U-q\mathbf {k}\right) \Phi \Phi ^{-1}\mathbf {k}\Phi \\&=\Phi ^{-1}U^{-1}\left( p\frac{\partial }{\partial \alpha }U+q\mathbf {k}U-qU\mathbf {k}\right) \mathbf {k}\Phi ,\quad \text {and}\\ n_1^\alpha (x_1^\alpha -x^\alpha )&=\Phi _1^{-1}\mathbf {k}\Phi _1\Phi ^{-1}\left( pU^{-1}\frac{\partial }{\partial \alpha }U+qU^{-1}\mathbf {k}U-q\mathbf {k}\right) \Phi \\&=\Phi ^{-1}U^{-1}\mathbf {k}\left( p\frac{\partial }{\partial \alpha }U+q\mathbf {k}U-qU\mathbf {k}\right) \Phi . \end{aligned}$$

Therefore, ${\text {tr}}((x_1^\alpha -x^\alpha )(n^\alpha +n_1^\alpha ))=0\iff {\text {tr}}(U^{-1}(\frac{\partial }{\partial \alpha }U\mathbf {k}+\mathbf {k}\frac{\partial }{\partial \alpha }U))=0$, which is equivalent to $U_{22}\frac{\partial }{\partial \alpha }U_{11}=U_{11}\frac{\partial }{\partial \alpha }U_{22}$.

To complete the proof we show that ${\text {tr}}(\partial x^\alpha \,n^\alpha )=p{\text {tr}}(\frac{\partial }{\partial \alpha }V\mathbf {k})$. Since $\Vert n^\alpha \Vert ^2=-\frac{1}{2}{\text {tr}}(n^\alpha \,n^\alpha )=1$, we have ${\text {tr}}(\partial n^\alpha n^\alpha )=0$. Moreover,

$$\begin{aligned} \partial \left( \Phi ^{-1}\frac{\partial }{\partial \alpha }\Phi \right)&=\partial \left( \Phi ^{-1}\right) \frac{\partial }{\partial \alpha }\Phi +\Phi ^{-1}\frac{\partial ^2}{\partial t\partial \alpha }\Phi =\left( V\Phi \right) ^H\frac{\partial }{\partial \alpha }\Phi +\Phi ^{-1}\frac{\partial }{\partial \alpha }\left( V\Phi \right) \\&=\Phi ^{-1}V^H\frac{\partial }{\partial \alpha }\Phi +\Phi ^{-1}\frac{\partial }{\partial \alpha }(V)\Phi +\Phi ^{-1}V\frac{\partial }{\partial \alpha }\Phi =\Phi ^{-1}\frac{\partial }{\partial \alpha }(V)\Phi , \end{aligned}$$

where we have used that $\Phi ^{-1}=\Phi ^H$ and that $V^H+V=0$. Hence, $\partial x^\alpha \perp n^\alpha \iff {\text {tr}}(\frac{\partial }{\partial \alpha }V\mathbf {k})=0$. $\square$

#### Corollary 4

The semidiscrete surfaces from Definition 12 are coupled.

#### Proof

We have $U_{22}\frac{\partial }{\partial \alpha }U_{11}=U_{11}\frac{\partial }{\partial \alpha }U_{22}=|a|^2\frac{\partial }{\partial \alpha }(\frac{1}{\gamma })$ and $\frac{\partial }{\partial \alpha }V_{11}=\frac{\partial }{\partial \alpha }V_{22}=0$. $\square$

The main result of the present section is that the surfaces $(\check{x}^\alpha , n^\alpha )$ and $(\hat{x}^\alpha , n^\alpha )$ have constant mean curvature in the sense of Definition 4.

#### Theorem 2

Let $(\check{x}^\alpha ,n^\alpha )$ and $(\hat{x}^\alpha ,n^\alpha )$ be given as in Definition 12. Then, for every $\alpha \in \mathbb {R}$, we have $A(\check{x}^\alpha ,\hat{x}^\alpha )=0$ throughout the parameter domain.

#### Proof

The statement can be verified by direct computations. However, since the involved expressions are rather lengthy, we defer the proof to the Appendix. $\square$

#### Corollary 5

The semidiscrete surfaces $(\check{x}^\alpha ,n^\alpha )$ and $(\hat{x}^\alpha ,n^\alpha )$ from Definition 12 have constant mean curvatures $\check{H}=1$ and $\hat{H}=-1$, respectively. The central surface $(x^\alpha ,n^\alpha )$ has constant Gauss curvature $K=4$.

Just like in the smooth case, the surfaces $(\check{x}^0,n^0)$ and $(\hat{x}^\alpha , n^\alpha )$ turn out to be isothermic and dual to each other.

#### Lemma 13

Consider the families $(\check{x}^\alpha ,n^\alpha )$, $(x^\alpha ,n^\alpha )$, and $(\hat{x}^\alpha ,n^\alpha )$ from Definition 12. Then, for $j\in \mathbb {Z}$, we have

$$\begin{aligned} Q\left[ \check{x}^{j\frac{\pi }{2}}\right]&=Q\left[ \hat{x}^{j\frac{\pi }{2}}\right] =-\frac{\gamma ^2}{\delta ^2},\\ Q\left[ x^{j\frac{\pi }{2}}\right]&=-\frac{\gamma ^2}{\delta ^2}\frac{\left( v-(-1)^{j}v^{-1}\right) \left( v_1-(-1)^{j}v_1^{-1}\right) }{\left( u+(-1)^{j}u^{-1}\right) ^2},\\ Q\left[ n^{j\frac{\pi }{2}}\right]&=-\frac{\gamma ^2}{\delta ^2}\frac{\left( v+(-1)^{j}v^{-1}\right) \left( v_1+(-1)^{j}v_1^{-1}\right) }{\left( u-(-1)^{j}u^{-1}\right) ^2}. \end{aligned}$$

#### Proof

Inserting the respective expressions derived in the proof of Theorem 2 into the formula for the cross ratio (cf. Definition 9) immediately yields the stated results. $\square$

#### Corollary 6

For every fixed $j\in \mathbb {Z}$, the semidiscrete surfaces $(\check{x}^{j\frac{\pi }{2}}, n^{j\frac{\pi }{2}})$ and $(\hat{x}^{j\frac{\pi }{2}}, n^{j\frac{\pi }{2}})$ are isothermic and dual to each other.

#### Proof

Isothermicity immediately follows from the previous lemma (cf. also Lemma 5). Duality is a consequence of Theorem 2. $\square$

## Semidiscrete Delaunay surfaces and elliptic billiards

In this section we construct semidiscrete cmc surfaces of rotational symmetry with discrete profile curves. To this purpose we assume that the Lax matrices *U* and *V* of the form (7) are independent of the continuous parameter *t*. In this case, the compatibility condition (8) resp. the Eq. (9) are given by

$$\begin{aligned} \delta = \text {const.},\quad u^2 = vv_1,&\quad {\text {Im}}(a)=0,\\ (b_1 - b){\text {Re}}(a) = uv + uv_1 - \frac{1}{uv} - \frac{1}{uv_1},&\text {~~and~~}(b_1+b)u={\text {Re}}(a)(v-v_1). \end{aligned}$$

To resolve the relation $u^2=vv_1$, we introduce a function $w:\mathbb {Z}\rightarrow \mathbb {R}_+$ and set $v=w^2$ and $u=ww_1$. Next we try to solve the resulting equations

$$\begin{aligned} (b_1\!-\!b){\text {Re}}(a)\!=\!w^3w_1\!+\!ww_1^3\!-\!\frac{1}{w^3w_1}\!-\!\frac{1}{ww_1^3},\ (b_1\!+\!b)ww_1={\text {Re}}(a)(w^2-w_1^2) \end{aligned}$$

for the successors $b_1$ and $w_1$ of *b* and *w*, respectively. From the equation on the right hand side we get $b_1={\text {Re}}(a)\frac{w^2-w_1^2}{ww_1}-b$. Inserting this expression into the left hand equation yields the following condition for $w_1$:

$$\begin{aligned} w^4w_1^6+\left( w^6+{\text {Re}}(a)^2w^2 \right) w_1^4+2{\text {Re}}(a)bw^3w_1^3- \left( {\text {Re}}(a)^2w^4+1 \right) w_1^2-w^2=0. \end{aligned}$$

Due to Descartes’ rule of signs there exists a unique positive solution $w_1$ of the latter equation.

Hence, for any given sequence $a:\mathbb {Z}\rightarrow \mathbb {R}$ and initial values $w(0)\in \mathbb {R}_+$, $b(0)\in \mathbb {R}$, the values *w*(*k*) and *b*(*k*) can be determined recursively for all $k\in \mathbb {Z}_+$. In this way we obtain Lax matrices $U(k,\alpha )$ and $V(k,\alpha )$ of the form (7) fulfilling the compatibility condition (8). Consequently, there exists a solution $\Phi =\Phi (k,t,\alpha )$ of the corresponding system (6). Given that $\Phi _1=U\Phi$ and $\Phi (0,0,\alpha )=\mathbf {1}$, we have

$$\begin{aligned} \Phi (k,0,\alpha )=U(k-1,\alpha )U(k-2,\alpha )\cdots U(1,\alpha )U(0,\alpha ). \end{aligned}$$

Solving $\partial \Phi =V\Phi$ finally yields

$$\begin{aligned} \Phi (k,t,\alpha )=\exp \left( V(k,\alpha )t \right) \Phi (k,0,\alpha ). \end{aligned}$$

**Fig. 3:**
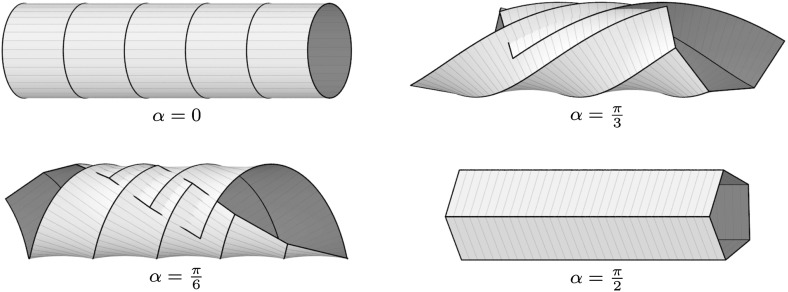
A semidiscrete cylinder with radius $r=1/2$ and three members of its associated family. The parameters $a=\text {const.}=2\sqrt{1+\frac{2}{\sqrt{5}}}$, $w(0)=1$, $b(0)=0$, and $\delta =4$ have been chosen such that we get a periodic surface for $\alpha =\pi /2$

**Fig. 4:**
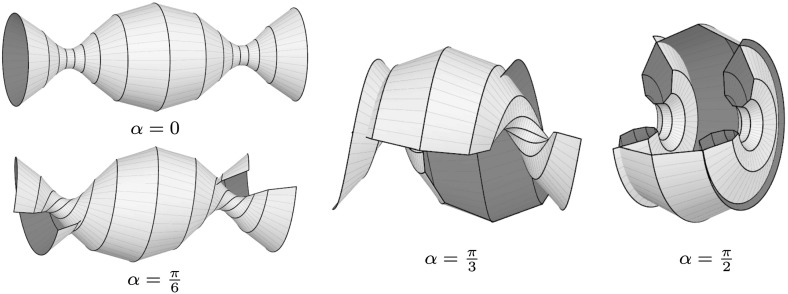
A semidiscrete unduloid (*top left*) and three members of its associated family. The corresponding parameters are $a=\text {const.}\approx 6.29$, $w(0)=1$, $b(0)=2$, and $\delta =4\sqrt{5}$. For $\alpha =\pi /2$ we obtain a semidiscrete nodoid (*right*)

By inserting this frame into the Sym–Bobenko formula (see Definition 12), we obtain semidiscrete Delaunay surfaces together with their associated families. For example, the initial values $w(0)=1$ and $b(0)=0$ yield $w(k)=1$ and $b(k)=0$ for all $k\in \mathbb {Z}_+$. The corresponding surfaces are semidiscrete cylinders with radius $r=1/2$ (see Fig. 3). By choosing $b(0)\ne 0$, we obtain more general semidiscrete Delaunay surfaces (see Fig. 4).

**Fig. 5:**
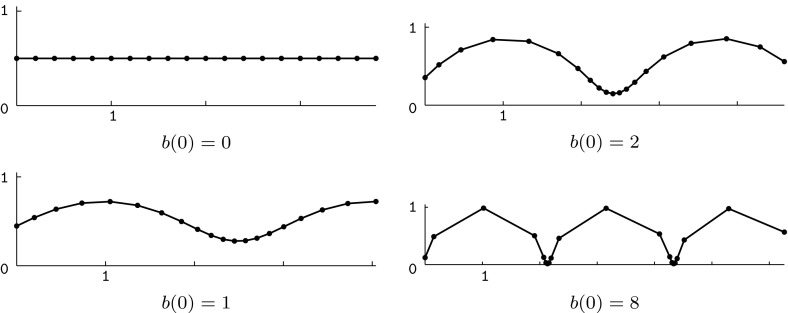
Profile curves of semidiscrete rotational symmetric cmc surfaces for different choices of the initial value *b*(0), which controls the oscillation of the meridean polygon. Here, $\alpha =0$, $a=\text {const.}=10$, and $w(0)=1$

**Fig. 6:**
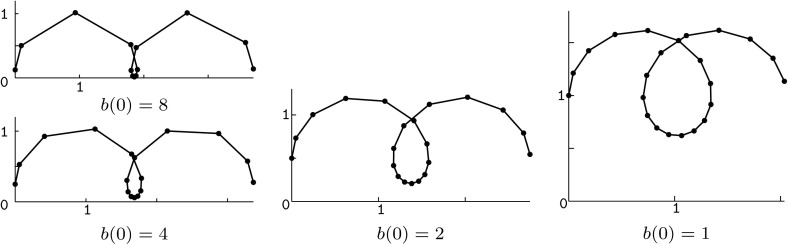
Profile curves of semidiscrete rotational symmetric cmc surfaces for different choices of the initial value *b*(0). Here, $\alpha =\pi /2$, $a=\text {const.}=10$, and $w(0)=1$

Observe that the initial value *b*(0) regulates the shape of the profile curve of the isothermic rotational symmetric cmc surface gained for $\alpha =0$. Setting $b(0)=0$ yields a straight line and in the limit $b(0)\rightarrow \infty$ we end up with consecutive half circles (cf. Fig. 5). The resulting surfaces are semidiscrete unduloids. Simultaneously, for $\alpha =\pi /2$, we obtain the profile curves of semidiscrete nodoids (cf. Fig. 6).

**Fig. 7:**
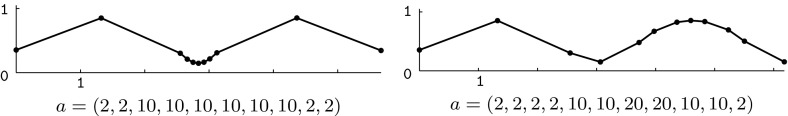
Profile curves of semidiscrete rotational symmetric cmc surfaces for different values of the sequence *a*, which controls the step size of the polygon. Here, $\alpha =0$, $w(0)=1$, and $b(0)=2$

Similarly, the sequence $a:\mathbb {Z}\rightarrow \mathbb {R}$ can be used to regulate the spacing between the vertices of the profile polygons. More precisely, the value *a*(*k*) is inversely proportional to the length of the edge $[\check{x}^0(k,t),\check{x}^0(k+1,t)]$. For an illustration see Fig. 7.

**Fig. 8:**
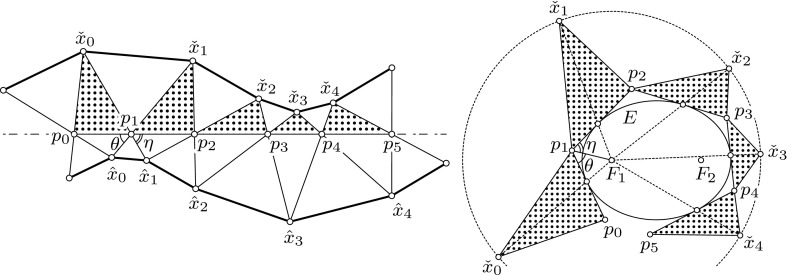
*Left*: Profile curves of dual semidiscrete rotational symmetric cmc surfaces $\check{x}$, $\hat{x}$. *Right*: An external elliptic billiard $\{p_k\}_{k\in \mathbb {Z}}$. Each dotted triangle $\{\check{x}_k, p_k, p_{k+1}\}$ on the *left* is mapped isometrically to the corresponding triangle on the *right*. However, for illustrational reasons, the figure on the *right* hand side has been scaled up uniformly

It turns out that there exists a nice geometric construction of the profile curves of semidiscrete rotational symmetric cmc surfaces. In fact, the discrete version of the classical Delaunay rolling ellipse construction for cmc surfaces of revolution described by Bobenko et al. [6, § 7.3] also applies to the semidiscrete setting (cf. Fig. 8). This has to be so, since for discrete surfaces of rotational symmetry the mean curvature is independent of the angle of rotation (see Bobenko et al. [6, § 7.2]). Thus, the notions of discrete and semidiscrete mean curvatures coincide in this particular case. Observe that, if the vertices $\{p_k\}_{k\in \mathbb {Z}}$ of the trajectory of the external elliptic billiard around *E* lie on an ellipse $E'$ confocal with *E*, then we have a classical reflection billiard in the ellipse $E'$, and the above construction agrees with that of Hoffmann [11]. For a comprehensive overview of mathematical billiards we refer to Tabachnikov [22].

## Appendix: Proof of Theorem 2

Here we provide the proof of Theorem 2. We show that the coupled semidiscrete surfaces $(\check{x}^\alpha , n^\alpha )$ and $(\hat{x}^\alpha ,n^\alpha )$ from Definition 12 satisfy $A(\check{x}^\alpha ,\hat{x}^\alpha )=0$. Applying the Binet–Cauchy identity to the determinants occurring in the mixed area form yields

10 $$\begin{aligned} A(\check{x}^\alpha ,\hat{x}^\alpha )=0 \iff&\langle \Delta \check{x}^\alpha , \Delta n^\alpha \rangle \langle \partial \hat{x}^\alpha +\partial \hat{x}_1^\alpha ,\partial n^\alpha +\partial n_1^\alpha \rangle \nonumber \\&-\langle \Delta \check{x}^\alpha , \partial n^\alpha +\partial n_1^\alpha \rangle \langle \partial \hat{x}^\alpha +\partial \hat{x}_1^\alpha ,\Delta n^\alpha \rangle \nonumber \\&+\langle \Delta \hat{x}^\alpha , \Delta n^\alpha \rangle \langle \partial \check{x}^\alpha +\partial \check{x}_1^\alpha ,\partial n^\alpha +\partial n_1^\alpha \rangle \nonumber \\&-\langle \Delta \hat{x}^\alpha , \partial n^\alpha +\partial n_1^\alpha \rangle \langle \partial \check{x}^\alpha +\partial \check{x}_1^\alpha ,\Delta n^\alpha \rangle = 0. \end{aligned}$$

Moreover, we observe that, for every coupled semidiscrete surface (*x*, *n*), we have

$$\begin{aligned} \langle \Delta x, \partial n+\partial n_1\rangle =\langle \partial x,n_1\rangle -\langle \partial x_1,n\rangle =\langle \partial x+\partial x_1,\Delta n\rangle . \end{aligned}$$

Now, direct computations yield

$$\begin{aligned} \Delta \check{x}^\alpha&=\frac{1}{\gamma ^2}\Phi ^{-1}\begin{pmatrix} -i(2u^{-2}-\lambda ^2-\lambda ^{-2}) &{}\quad -2\bar{a}\frac{1}{u\lambda }\\ 2a\frac{\lambda }{u} &{}\quad i(2u^{-2}-\lambda ^2-\lambda ^{-2}) \end{pmatrix}\Phi ,\\ \partial \check{x}^\alpha&=\frac{2}{v\delta }\Phi ^{-1} \begin{pmatrix} 0 &{}\quad i\lambda ^{-1}\\ i\lambda &{}\quad 0 \end{pmatrix}\Phi ,\quad \partial \check{x}^\alpha _1=\frac{2}{v_1\delta _1}\Phi ^{-1}U^{-1} \begin{pmatrix} 0 &{}\quad i\lambda ^{-1}\\ i\lambda &{}\quad 0 \end{pmatrix}U\Phi , \end{aligned}$$

and

$$\begin{aligned} \Delta \hat{x}^\alpha&=\frac{1}{\gamma ^2}\Phi ^{-1}\begin{pmatrix} i(2u^2-\lambda ^2-\lambda ^{-2}) &{}\quad -2\bar{a}u\lambda \\ 2a\frac{u}{\lambda } &{}\quad -i(2u^2-\lambda ^2-\lambda ^{-2}) \end{pmatrix}\Phi ,\\ \partial \hat{x}^\alpha&=-\frac{2v}{\delta }\Phi ^{-1} \begin{pmatrix} 0 &{}\quad i\lambda \\ i\lambda ^{-1} &{}\quad 0 \end{pmatrix}\Phi ,\quad \partial \hat{x}^\alpha _1=-\frac{2v_1}{\delta _1}\Phi ^{-1}U^{-1} \begin{pmatrix} 0 &{}\quad i\lambda \\ i\lambda ^{-1} &{}\quad 0 \end{pmatrix}U\Phi . \end{aligned}$$

Further, for the Gauss map $n^\alpha$, we obtain

$$\begin{aligned} n^\alpha&= \Phi ^{-1}\mathbf {k}\Phi =\Phi ^{-1}\begin{pmatrix} -i &{}\quad 0 \\ 0 &{}\quad i \end{pmatrix}\Phi ,\quad \partial n^\alpha =-\frac{2i}{\delta }\Phi ^{-1} \begin{pmatrix} 0 &{}\quad v\lambda +\frac{1}{v\lambda }\\ \frac{\lambda }{v}+\frac{v}{\lambda } &{}\quad 0 \end{pmatrix}\Phi ,\\ n_1^\alpha&= \frac{1}{\gamma ^2}\Phi ^{-1}\begin{pmatrix} -i|a|^2-i\left( u\lambda -\frac{1}{u\lambda }\right) \!\left( \frac{\lambda }{u}-\frac{u}{\lambda }\right) &{}\quad -2\bar{a}\left( u\lambda -\frac{1}{u\lambda }\right) \\ 2a\left( \frac{u}{\lambda }-\frac{\lambda }{u}\right) &{}\quad i|a|^2+i\left( \frac{\lambda }{u}-\frac{u}{\lambda }\right) \!\left( u\lambda -\frac{1}{u\lambda }\right) \end{pmatrix}\Phi ,\\ \Delta n^\alpha&=\frac{2}{\gamma ^2}\Phi ^{-1}\begin{pmatrix} i\left( u^2+u^{-2}-\lambda ^2-\lambda ^{-2}\right) &{}\quad \bar{a}\left( \frac{1}{u\lambda }-u\lambda \right) \\ a\left( \frac{u}{\lambda }-\frac{\lambda }{u}\right) &{}\quad -i\left( u^2+u^{-2}-\lambda ^2-\lambda ^{-2}\right) \\ \end{pmatrix}\Phi ,\\ \partial n_1^\alpha&=-\frac{2i}{\delta _1}\Phi ^{-1}U^{-1} \begin{pmatrix} 0 &{}\quad v_1\lambda +\frac{1}{v_1\lambda }\\ \frac{\lambda }{v_1}+\frac{v_1}{\lambda } &{}\quad 0 \end{pmatrix}U\Phi . \end{aligned}$$

Next we compute

$$\begin{aligned} U^{-1}\!\begin{pmatrix} 0 &{} \quad i\lambda ^{-1} \\ i\lambda &{} \quad 0 \end{pmatrix}\! U&=\frac{1}{\gamma ^2}\!\begin{pmatrix} a\left( \frac{1}{u}-u\lambda ^2\right) -\bar{a}\left( \frac{1}{u}-\frac{u}{\lambda ^2}\right) &{}\quad i\frac{\bar{a}^2}{\lambda }+i\lambda \left( \frac{1}{u\lambda }-u\lambda \right) ^2\\ ia^2\lambda +\frac{i}{\lambda }\left( \frac{\lambda }{u}-\frac{u}{\lambda }\right) ^2 &{}\quad \!\bar{a}\left( \frac{1}{u}-\frac{u}{\lambda ^2}\right) -a\left( \frac{1}{u}-u\lambda ^2\right) \end{pmatrix}\!,\\ U^{-1}\!\begin{pmatrix} 0 &{}\quad \! i\lambda \\ i\lambda ^{-1} &{}\quad \! 0 \end{pmatrix}\! U&\quad \,{=}\,\frac{1}{\gamma ^2}\!\begin{pmatrix} a\left( \frac{1}{u\lambda ^2}-u\right) -\bar{a}\left( \frac{\lambda ^2}{u}-u\right) &{}\quad i\bar{a}^2\lambda +\frac{i}{\lambda }\left( \frac{1}{u\lambda }-u\lambda \right) ^2 \\ i\frac{a^2}{\lambda }+i\lambda \left( \frac{\lambda }{u}-\frac{u}{\lambda }\right) ^2 &{}\quad \!\bar{a}\left( \frac{\lambda ^2}{u}-u\right) -a\left( \frac{1}{u\lambda ^2}-u\right) \end{pmatrix}\!, \end{aligned}$$

and observe that

$$\begin{aligned} iU^{-1} \begin{pmatrix} 0 &{}\quad v_1\lambda +\frac{1}{v_1\lambda }\\ \frac{\lambda }{v_1}+\frac{v_1}{\lambda } &{}\quad 0 \end{pmatrix}U=U^{-1}\left[ v_1\begin{pmatrix} 0 &{}\quad i\lambda \\ i\lambda ^{-1} &{} \quad 0 \end{pmatrix}+\frac{1}{v_1}\begin{pmatrix} 0 &{}\quad i\lambda ^{-1} \\ i\lambda &{}\quad 0 \end{pmatrix}\right] U. \end{aligned}$$

Finally, we get

$$\begin{aligned}&\langle \Delta \check{x}^\alpha ,\Delta n^\alpha \rangle =\frac{-2}{\gamma ^2}\left( 2u^{-2}-\lambda ^2-\lambda ^{-2}\right) \!,\ \langle \Delta \hat{x}^\alpha ,\Delta n^\alpha \rangle =\frac{2}{\gamma ^2}\left( 2u^2-\lambda ^2-\lambda ^{-2}\right) \!,\\&\langle \partial \check{x}_1^\alpha ,n^\alpha \rangle =\frac{-4}{v_1\delta _1\gamma ^2}{\text {Im}}\left( a\left( u^{-1}-u\lambda ^{2}\right) \right) \!,\ \langle \partial \check{x}^\alpha ,\partial n^\alpha \rangle =\frac{-2}{\delta ^2}(2v^{-2}+\lambda ^2+\lambda ^{-2}),\\&\langle \partial \check{x}^\alpha ,n_1^\alpha \rangle =\frac{-4}{v\delta \gamma ^2}{\text {Im}}\left( a\left( u^{-1}-u\lambda ^{-2}\right) \right) \!,\ \langle \partial \check{x}_1^\alpha ,\partial n_1^\alpha \rangle =\frac{-2}{\delta _1^2}(2v_1^{-2}+\lambda ^2+\lambda ^{-2}),\\&\langle \partial \hat{x}_1^\alpha ,n^\alpha \rangle =\frac{-4v_1}{\delta _1\gamma ^2}{\text {Im}}\left( a\left( u-u^{-1}\lambda ^{-2}\right) \right) \!,\ \langle \partial \hat{x}^\alpha ,\partial n^\alpha \rangle =\frac{2}{\delta ^2}(2v^2+\lambda ^2+\lambda ^{-2}), \\&\langle \partial \hat{x}^\alpha ,n_1^\alpha \rangle =\frac{-4v}{\delta \gamma ^2}{\text {Im}}\left( a\left( u-u^{-1}\lambda ^2\right) \right) \!,\ \langle \partial \hat{x}_1^\alpha ,\partial n_1^\alpha \rangle =\frac{2}{\delta _1^2}(2v_1^2+\lambda ^2+\lambda ^{-2}), \end{aligned}$$

as well as

$$\begin{aligned} \langle \partial \check{x}^\alpha ,\partial n_1^\alpha \rangle&\,{=}\,\frac{-4}{v\delta \delta _1\gamma ^2}{\text {Re}}\left( a^2\!\left( v_1^{-1}+v_1\lambda ^{-2}\right) {+}\left( v_1+v_1^{-1}\lambda ^{-2}\right) \!\left( u^{-1}\lambda -u\lambda ^{-1}\right) ^2\right) \!,\\ \langle \partial \hat{x}^\alpha ,\partial n_1^\alpha \rangle&\,{=}\,\frac{4v}{\delta \delta _1\gamma ^2}{\text {Re}}\left( a^2\!\left( v_1+v_1^{-1}\lambda ^2\right) {+}\left( v_1^{-1}+v_1\lambda ^2\right) \!\left( u^{-1}\lambda -u\lambda ^{-1}\right) ^2\right) \!,\\ \langle \partial \check{x}_1^\alpha ,\partial n^\alpha \rangle&\,{=}\,\frac{-4}{v_1\delta \delta _1\gamma ^2}{\text {Re}}\left( a^2\!\left( v^{-1}+v\lambda ^2\right) {+}\left( v+v^{-1}\lambda ^{-2}\right) \!\left( u^{-1}\lambda -u\lambda ^{-1}\right) ^2\right) \!,\\ \langle \partial \hat{x}_1^\alpha ,\partial n^\alpha \rangle&\,{=}\,\frac{4v_1}{\delta \delta _1\gamma ^2}{\text {Re}}\left( a^2\!\left( v+v^{-1}\lambda ^{-2}\right) {+}\left( v^{-1}+v\lambda ^2\right) \!\left( u^{-1}\lambda -u\lambda ^{-1}\right) ^2\right) \!. \end{aligned}$$

We complete the proof by substituting these expressions into Eq. (10) and using the fact that $\delta _1=\delta$ and $u^2=vv_1$. $\square$

## References

[1] Bobenko AI (1991). Constant mean curvature surfaces and integrable equations. Russ. Math. Surv..

[2] Bobenko, A.I.: Surfaces in terms of $$2$$ matrices. Old and new integrable cases. In: Harmonic Maps and Integrable Systems, pp. 83–127. Vieweg (1994)

[3] Bobenko AI, Hoffmann T, Springborn BA (2006). Minimal surfaces from circle patterns: geometry from combinatorics. Ann. Math..

[4] Bobenko AI, Pinkall U (1996). Discrete isothermic surfaces. J. Reine Angew. Math..

[5] Bobenko, A.I., Pinkall, U.: Discretization of surfaces and integrable systems. In: Discrete Integrable Geometry and Physics, vol. 16, pp. 3–58. Oxford University Press, Oxford (1999)

[6] Bobenko AI, Pottmann H, Wallner J (2010). A curvature theory for discrete surfaces based on mesh parallelity. Math. Ann..

[7] Bobenko AI, Suris YB (2008). Discrete Differential Geometry: Integrable Struture.

[8] Burstall F, Hertrich-Jeromin U, Müller C, Rossman W (2016). Semi-discrete isothermic surfaces. Geom. Dedicata.

[9] Cieśliński J, Doliwa A, Santini PM (1997). The integrable discrete analogues of orthogonal coordinate systems are multi-dimensional circular lattices. Phys. Lett. A.

[10] Fujimori S, Kobayashi S, Rossman W (2005). Loop Group methods for constant mean curvature surfaces. Rokko Lect. Math..

[11] Hoffmann T, Hege HC, Polthier K (1998). Discrete rotational cmc surfaces and the elliptic billiard. Mathematical Visualization.

[12] Hoffmann, T., Sageman-Furnas, A.O., Wardetzky, M.: A discrete parametrized surface theory in $$\mathbb{R}^3$$. IMRN (2014, to appear). arXiv:1412.7293

[13] Karpenkov O, Wallner J (2014). On offsets and curvatures for discrete and semidiscrete surfaces. Beitr. Algebra Geom..

[14] Konopelchenko BG, Schief WK (1998). Three-dimensional integrable lattices in Euclidean spaces: conjugacy and orthogonality. Proc. R. Soc. Lond. A.

[15] Müller C (2015). Semi-discrete constant mean curvature surfaces. Math. Z..

[16] Müller C, Wallner J (2013). Semi-discrete isothermic surfaces. Results Math..

[17] Pedit F, Wu H (1995). Discretizing constant curvature surfaces via loop group factorizations: the discrete sine- and sinh-Gordon equations. J. Geom. Phys..

[18] Pottmann, H., Liu, Y., Wallner, J., Bobenko, A., Wang, W.: Geometry of multi-layer freeform structures for architecture. ACM Trans. Graph. **26**(3), 65:1–65:11 (2007)

[19] Pottmann, H., Schiftner, A., Bo, P., Schmiedhofer, H., Wang, W., Baldassini, N., Wallner, J.: Freeform surfaces from single curved panels. ACM Trans. Graph. **27**(3), 76:1–76:10 (2008)

[20] Rossman W, Yasumoto M (2012). Weierstrass representation for semi-discrete minimal surfaces, and comparison of various discretized catenoids. J. Math. Ind..

[21] Sym, A.: Soliton surfaces and their applications. In: Lecture Notes in Physics, vol. 239, pp. 154–231. Springer, Berlin (1985)

[22] Tabachnikov S (2005). Geometry and Billiards, Student Mathematical Library.

[23] Wallner J (2012). On the semidiscrete differential geometry of A-surfaces and K-surfaces. J. Geom..

